# Platinum Nanoparticles as Modulators of Idarubicin Activity: A Physicochemical and In Vitro Biological Study

**DOI:** 10.3390/ph19081274

**Published:** 2026-08-12

**Authors:** Marcin Zakrzewski, Patrycja Bełdzińska, Karolina Gackowska, Aliaksandra Yurchak, Marzena Jamrógiewicz, Dariusz Wyrzykowski, Katarzyna Bury, Katarzyna Grzyb, Grzegorz Gołuński, Jacek Piosik

**Affiliations:** 1 Laboratory of Biophysics, Intercollegiate Faculty of Biotechnology UG and MUG, University of Gdańsk, 80-307 Gdańsk, Poland; 2 Laboratory of Recombinant Vaccines, Intercollegiate Faculty of Biotechnology UG and MUG, University of Gdańsk, 80-307 Gdańsk, Poland; 3 Department of Physical Chemistry, Faculty of Pharmacy, Medical University of Gdańsk, 80-416 Gdańsk, Poland; majam@gumed.edu.pl; 4 Department of General and Inorganic Chemistry, Faculty of Chemistry, University of Gdańsk, 80-309 Gdańsk, Poland; 5 Laboratory of Molecular Biology, Intercollegiate Faculty of Biotechnology UG and MUG, University of Gdańsk, 80-307 Gdańsk, Poland; 6 Laboratory of Virus Molecular Biology, Intercollegiate Faculty of Biotechnology UG and MUG, University of Gdańsk, 80-307 Gdańsk, Poland

**Keywords:** platinum nanoparticles, idarubicin, leukaemia, mutagenicity, cytotoxicity, MCF-7, SK-BR-3

## Abstract

**Background/Objectives**: Cancer remains one of the leading causes of death worldwide. Although chemotherapy is widely used, it is associated with severe side effects, including myelosuppression and systemic toxicity. Nanoparticles have emerged as promising candidates for modulating drug activity. In this study, we investigated whether platinum nanoparticles (PtNPs) of various sizes interact with idarubicin (IDA), an anthracycline anticancer drug used primarily to treat acute leukaemia. **Methods**: Interactions between PtNPs and IDA were analysed using dynamic light scattering (DLS), atomic force microscopy (AFM), fluorescence spectroscopy, Fourier-transform infrared (FTIR) spectroscopy, and near-infrared (NIR) spectroscopy. Thermodynamic and thermal properties were assessed using isothermal titration calorimetry (ITC) and differential scanning calorimetry (DSC). Biological effects were evaluated using the Ames mutagenicity assay on the *Salmonella enterica* serovar Typhimurium TA98 strain and cytotoxicity assays on SK-BR-3 and MCF-7 cell lines. **Results**: DLS demonstrated changes in the hydrodynamic diameter of PtNPs following IDA addition, which were further supported by AFM imaging. PtNPs significantly quenched IDA fluorescence, indicating close molecular interactions, which were further supported by FTIR and NIR. ITC revealed that the interactions were endothermic, with enthalpy values ranging from 1.2 to 3.6 kcal/mol, and DSC demonstrated that the PtNP-IDA combination altered the melting temperature of IDA. Biological assays revealed that all examined PtNP sizes influenced IDA mutagenicity in the *Salmonella enterica* serovar Typhimurium TA98 strain. Furthermore, PtNPs modulated the cytotoxicity of IDA in SK-BR-3 and MCF-7 cell lines in a dose-dependent manner. **Conclusions**: These findings demonstrate that PtNPs interact with IDA and modulate its biological activity. However, in this study no non-cancerous cell lines were examined; therefore, the observed interactions and biological effects support further investigation of the PtNP-IDA combination in the context of nanoparticle-assisted anticancer therapy on a broader range of in vitro cell lines.

## 1. Introduction

In 2022, cancer accounted for approximately 10 million deaths worldwide, ranking among the ten leading causes of mortality [1,2]. Moreover, the incidence of this disease is projected to increase by approximately 70% by 2050 [1]. Despite the development of various novel treatment regimens, chemotherapy remains one of the most widely used treatment modalities [3,4]. However, its therapeutic efficacy is limited by its lack of selectivity towards healthy cells, resulting in a wide range of adverse effects, including bone marrow suppression and an increased risk of secondary malignancies [4]. Furthermore, cancer cells can develop drug resistance, rendering the therapy ineffective over time [3]. To address these limitations, current research focuses on modulating the activity of established anticancer agents by combining them with nanoparticles [5,6].

Nanomedicine, defined as the application of nanoscale materials for diagnostic and therapeutic purposes, has emerged rapidly over the last few decades [7]. In general, nanoparticles range from 1 to 100 nm in size and can be synthesised using various physical, chemical or biological methods, with biological approaches becoming increasingly common in recent years [8,9]. Advances in synthesis and characterisation techniques have enabled precise control over the size, shape, and surface chemistry, thus broadening their potential for clinical applications, such as therapy, diagnostics, or drug delivery [10]. Among the wide range of nanomaterials, metallic nanoparticles have gained significant interest over the years, especially due to their unique characteristics, including a high surface-area-to-volume ratio and both optical and catalytic properties. Within this group, platinum nanoparticles (PtNPs) have attracted considerable attention over the last decade due to diverse biomedical applications, including antibacterial and antifungal activity, biosensing, and bioimaging [11]. However, it is their anticancer potential that has subsequently emerged as one of their most extensively investigated properties. They have been shown to exert cytotoxicity against various cancerous cell lines [12,13,14], while minor or no cytotoxicity towards healthy tissue has been observed, indicating limited toxicity or possible protective properties [15,16]. PtNPs exert their biological effects through multiple mechanisms; however, the predominant mode of action is generally attributed to reactive oxygen species (ROS) generation [12,17]. Furthermore, PtNPs have been shown to accumulate strongly in tumours. This phenomenon may be attributed to the enhanced permeability and retention (EPR) effect, which results from leaky blood vessels and leads to increased passive uptake of macromolecules [18]. Due to this, PtNPs have been applied as nanocarriers, leading to a more selective drug release at the tumour site [19,20,21,22]. Moreover, PtNPs provide a versatile platform for surface functionalization with diverse ligands, enabling broader applications, thus making them an excellent tool for future modulations. However, since the main cytotoxic effect is generally attributed to the generation of ROS, the toxicological evidence remains inconsistent. While some studies have reported protective effects towards healthy cells, others have depicted opposite effects. In addition to that, accumulation of nanoparticles in organs such as the spleen, liver or kidneys is a known problem, and the consequences of prolonged exposure still need to be fully elucidated [23,24]. Therefore, further studies are needed to evaluate both the therapeutic potential and the safety of PtNP-drug combinations.

Idarubicin (IDA) is an anthracycline active pharmaceutical ingredient (API) used in anticancer therapy. It belongs to the anthracycline class of antibiotics and is a semisynthetic derivative of daunorubicin (DAU), with a methoxy group replaced by hydrogen at the R_1_ position [25]. This seemingly small substitution renders IDA more lipophilic, improving membrane penetration [26,27]. Similar to other anthracyclines, IDA acts through intercalation into DNA, inhibition of topoisomerase II activity, and generation of free radicals [28,29]. Therefore, it is primarily used in the treatment of acute myeloid and lymphoblastic leukaemia, including in combination with cytarabine (CYT) [27], demonstrating greater efficacy than the DAU-CYT combination [30]. Unfortunately, the use of this API may cause acute side effects, such as myelosuppression and cardiotoxicity [28,31]. Nevertheless, its efficacy has justified its widespread clinical use.

The available evidence concerning the potential role of nanoparticles in cancer chemotherapy remains limited, emphasising the necessity for additional comprehensive research. In our previous studies on the influence of PtNPs on DAU’s activity, we observed significant aggregation resulting in fluorescence quenching and changes in the chemical environment as evidenced by spectroscopic methods, as well as decreased mutagenicity towards *Salmonella enterica* serovar Typhimurium TA98. However, no significant effect of PtNPs was observed on DAU cytotoxicity in MelJuSo and HaCaT cell lines. Based on these findings and considering that PtNPs have demonstrated a multitude of anticancer effects on their own and in combination with cytotoxic agents in other systems, we therefore investigated whether spherical PtNPs of distinct sizes (5, 30, 50, and 70 nm) can potentially alter the biological activity of IDA. We hypothesised that such a combination would result in changes in the physicochemical characteristics of the drug, as well as its biological efficacy. Such a putative idarubicin-PtNP system requires comprehensive physicochemical characterisation, including evaluation of intermolecular interactions and aggregation behaviour, together with assessment of its biological activity.

## 2. Results and Discussion

Metallic nanoparticles exhibit a broad range of physicochemical properties, making them promising platforms for biomedical applications. Extensive research has investigated the potential of gold [32], silver [33], or iron nanoparticles [34], demonstrating their significant antimicrobial and often also anticancer effects. Moreover, metallic nanoparticles have also been widely employed in both bioimaging and drug delivery systems (DDSs) [35,36]. Considering their broad biomedical potential, this study aimed to investigate the interactions between platinum nanoparticles (PtNPs) of distinct sizes (5, 30, 50, and 70 nm) and idarubicin (IDA), as well as the impact of these interactions on the biological activity of IDA.

### 2.1. PtNPs Aggregate with IDA

**Figure 1:**
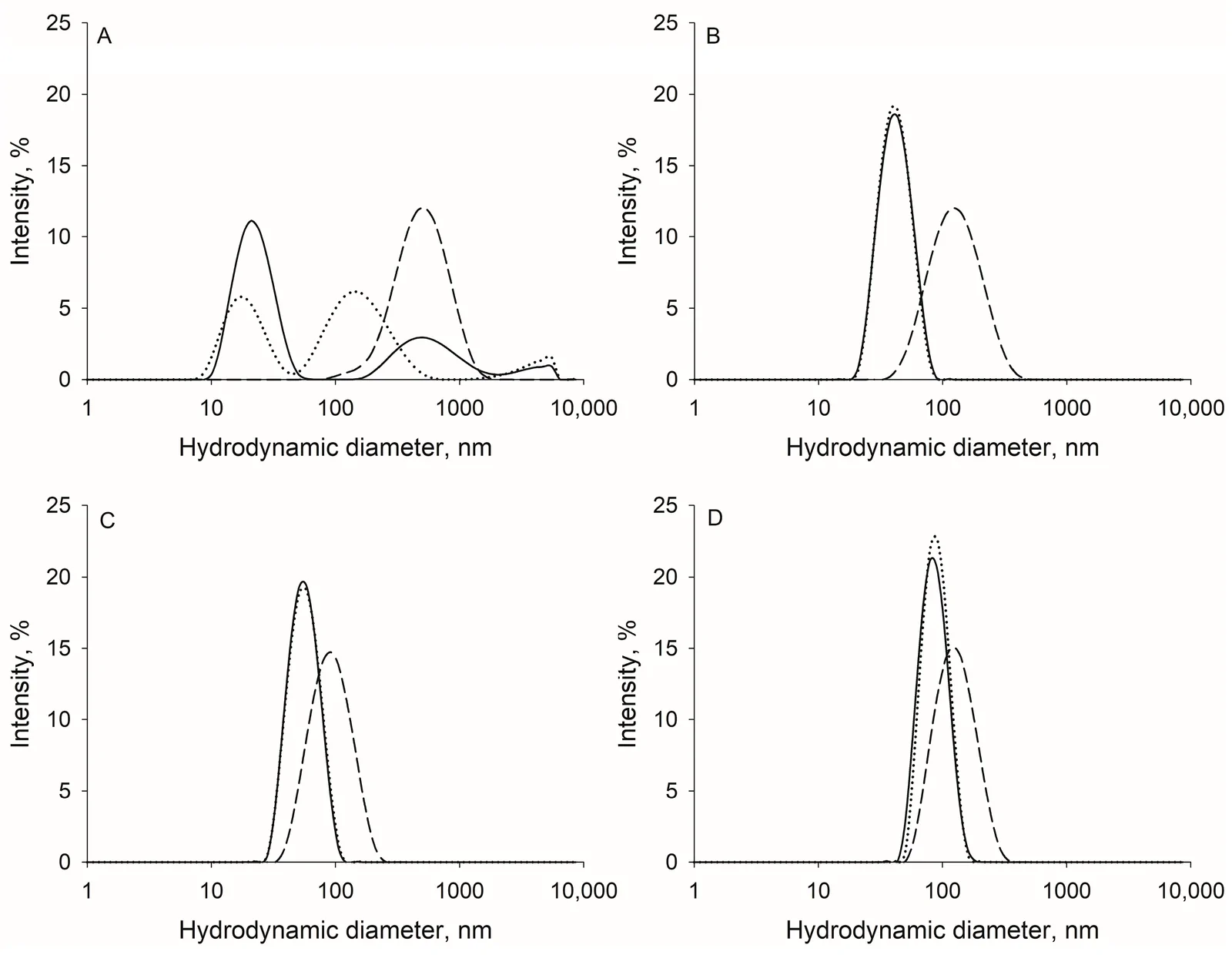
**Aggregation pattern of platinum nanoparticles (PtNPs) with idarubicin (IDA).** (**A**) 5 nm PtNPs, (**B**) 30 nm PtNPs, (**C**) 50 nm PtNPs, (**D**) 70 nm PtNPs. Solid line—nanoparticles alone; dotted line—nanoparticles with the addition of 5 µM IDA; dashed line—nanoparticles with the addition of 50 µM IDA.

Firstly, we employed dynamic light scattering (DLS) to assess potential interactions between PtNPs and IDA (Figure 1). The nanoparticles were of identical diameter as stated by the manufacturer (Table S1). The 5 nm PtNPs exhibited a broad size distribution, with the predominant fraction displaying an average diameter of approximately 23 ± 1.3 nm. The addition of 5 µM of IDA resulted in a noticeable increase in hydrodynamic diameter. At an IDA concentration of 50 µM, the population became narrower, as reflected by the decrease in the polydispersity index (PdI) from 0.5 to 0.24, while the mean hydrodynamic diameter increased further to 539.7 ± 16 nm. In the case of 30, 50, and 70 nm PtNPs, we observed a significant change in registered diameters only in combination with 50 µM of IDA, while all the PdI values remained below 0.3. Detailed data are provided in the Supplementary Information (Table S2). These results indicate the possible formation of PtNP-IDA heteroaggregates. However, an alternative explanation is that IDA may promote the aggregation of PtNPs themselves, leading to the formation of homoaggregates of diverse dimensions rather than heteroaggregates. To fully elucidate this mechanism, further studies are required.

**Figure 2:**
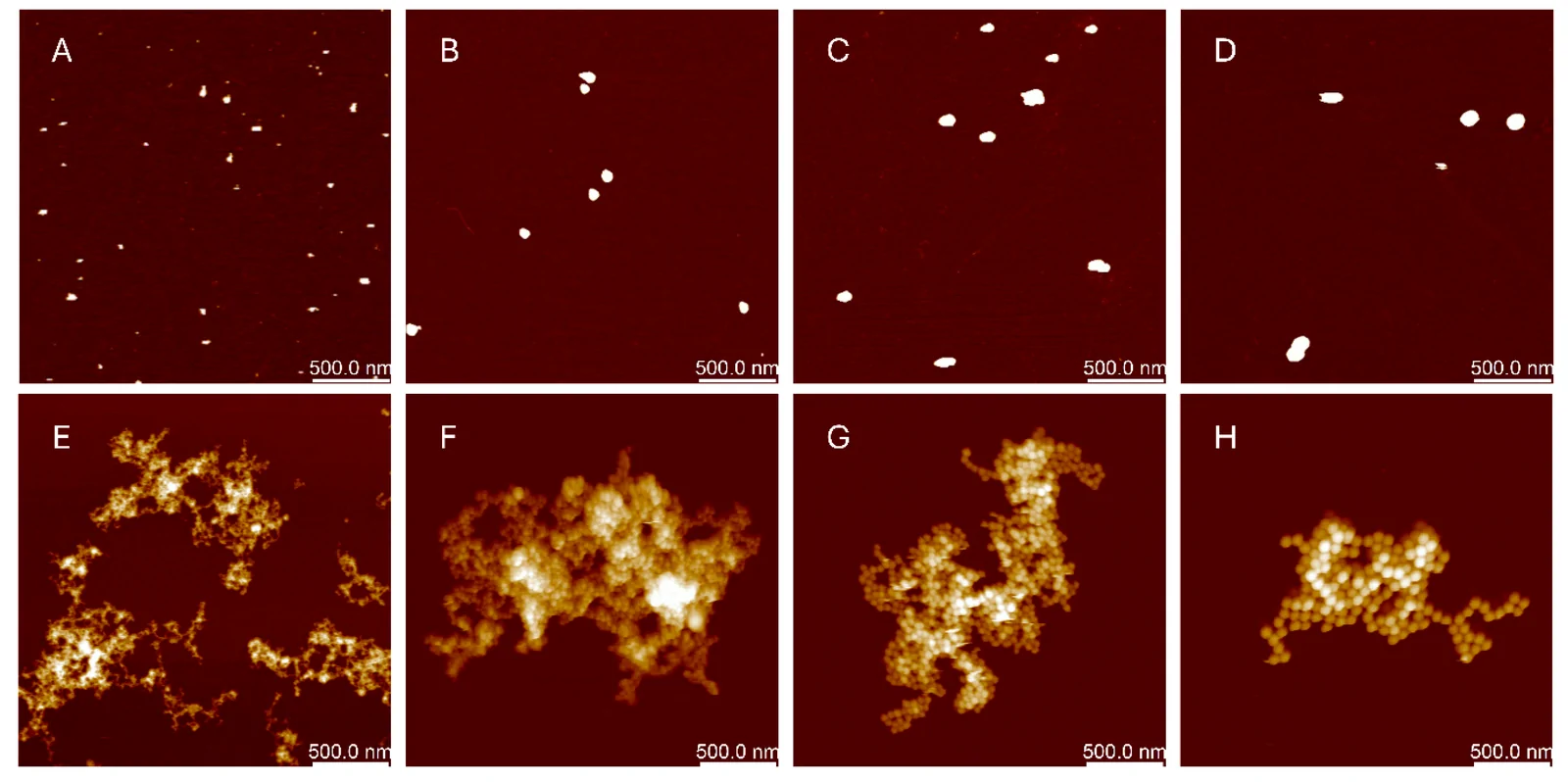
**Atomic force microscopy (AFM) pictures of platinum nanoparticles (PtNPs) alone and in combination with idarubicin (IDA).** (**A**–**D**) 5, 30, 50, and 70 nm PtNPs, respectively; (**E**–**H**) 5, 30, 50, and 70 nm PtNPs with IDA, respectively.

Furthermore, we evaluated the formation of PtNP-IDA aggregates using atomic force microscopy (AFM) (Figure 2). All tested PtNPs sizes were visualised as individual particles or very small clusters. With the addition of IDA, we observed the formation of aggregates in all the samples, and the mean registered size increased 225-, 46-, 21-, and 20-fold for 5, 30, 50, and 70 nm PtNPs, respectively (Table S3). The aggregates varied considerably in size and morphology, further suggesting that IDA may promote the formation of PtNP-IDA heteroaggregates, although the formation of PtNP homoaggregates cannot be excluded.

Overall, the AFM and DLS are consistent with our previous studies, where PtNPs were found to aggregate with doxorubicin (DOX) [37], epirubicin (EPI) [38] and daunorubicin (DAU) [39], as well as with other research group studies on PtNP-DOX [40] and AuNP-DOX [41]. However, the differences in the magnitude of increase in registered size between these two methods may come from several factors. Firstly, DLS measures the hydrodynamic diameter of particles suspended in a solution, whereas AFM characterizes dried samples immobilized on a mica surface. Therefore, the sample deposition and solvent evaporation can promote additional clustering through particle concentration and capillary forces [42]. Secondly, these techniques probe different physical properties and exhibit different detection biases; DLS reports an intensity-weighed hydrodynamic size distribution, and AFM directly images the deposited aggregates. Consequently, quantitative differences between these aggregates may arise. Nevertheless, the results of these methods combined strongly suggest that while nanoparticles alone were predominantly not aggregated, the addition of IDA promoted this process. These aggregates may consist of PtNP-IDA heteroaggregates and/or PtNP homoaggregates, and the contribution of each type cannot be unanimously distinguished based on the present data.

### 2.2. PtNPs Interact with IDA

**Figure 3:**
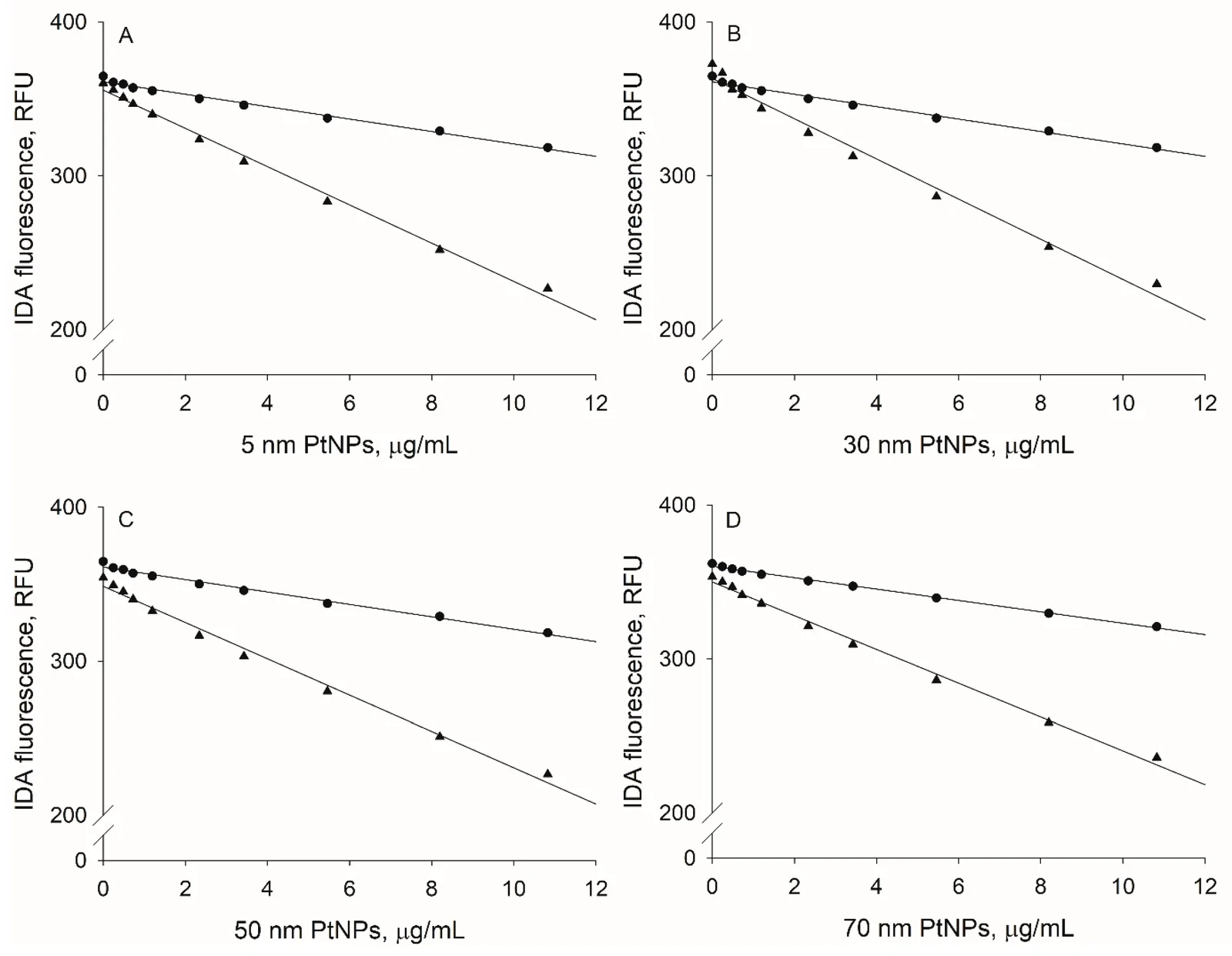
**Platinum nanoparticle (PtNP) influence on idarubicin’s (IDA) fluorescence at 545 nm.** (**A**) 5 nm PtNPs, (**B**) 30 nm PtNPs, (**C**) 50 nm PtNPs, (**D**) 70 nm PtNPs. Dots—negative control (IDA with sodium citrate); triangles—IDA with PtNPs.

To further establish the nature of interactions between PtNPs and IDA, we utilised IDA’s fluorescence capabilities and performed fluorescence spectroscopy (Figure 3). PtNPs of all tested sizes caused a significant decrease in the fluorescence intensity of IDA. Conversely, titration with identical volumes of appropriate sodium citrate solution did not exhibit comparable changes. The complete fluorescence spectra are provided in the Supplementary Information (Figure S1).

Metallic nanoparticles are known to exhibit, among other properties, a metal-enhanced fluorescence (MEF) effect. In principle, they can increase the emission intensity of nearby fluorophores at an optimal distance. However, when the distance between the fluorophore and the nanoparticle surface decreases below 5 nm, fluorescence quenching may occur, likely due to interactions with the nanoparticle surface and excitation of localised surface plasmon resonance (SPR) [43,44]. The observed decrease in fluorescence intensity is therefore consistent with IDA molecules approaching the PtNP surface sufficiently closely for nanoparticle-mediated fluorescence attenuation to occur. However, the fluorescence measurements were based solely on emission intensity and were not corrected for scattering, absorbance, or inner-filter effects. Therefore, the present data does not allow for unequivocal distinction between true molecular quenching and possible nanoparticle-induced optical effects. Nevertheless, the absence of a comparable effect in the sodium citrate controls supports the conclusion that the observed fluorescence quenching is associated with the presence of PtNPs. Analogous effects were observed in PtNP-DOX [37,45], PtNP-EPI [38] and PtNP-DAU [39] analyses, as well as in other studies on gold, silver and iron nanoparticles [46,47,48].

**Figure 4:**
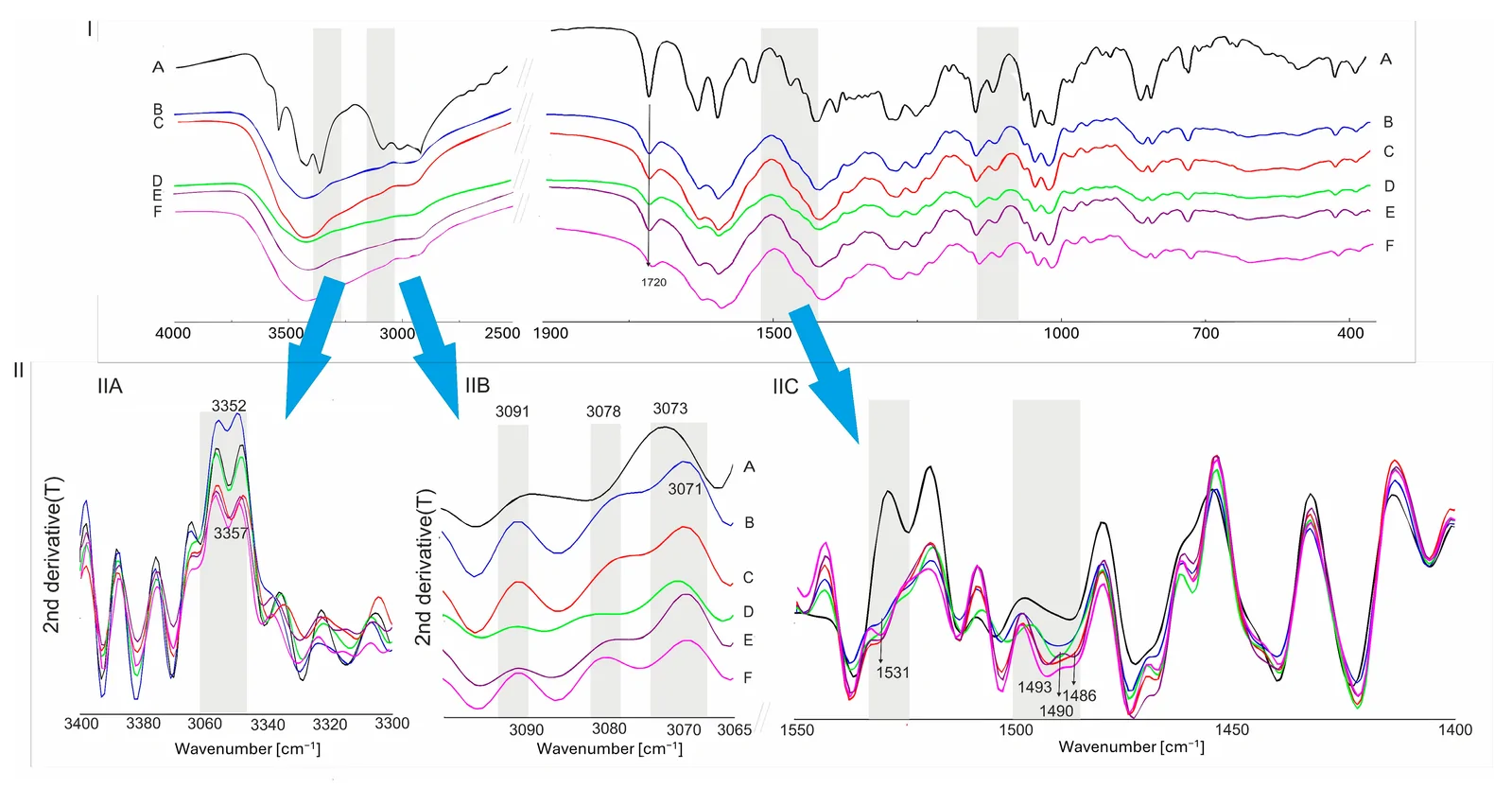
**Fourier-transform infrared spectroscopy (FTIR) spectra of the investigated combinations of idarubicin (IDA) and platinum nanoparticles (PtNPs) (I) at regions 4000–2500 cm^−1^ and 1900–400 cm^−1^, and (II) their second derivatives.** (A) Black—IDA; (B) blue—IDA lyophilised from sodium citrate; (C) red—5 nm PtNPs with IDA; (D) green—30 nm PtNPs with IDA; (E) violet—50 nm PtNPs with IDA; (F) pink—70 nm PtNPs with IDA. Gray areas—regions of significant spectral changes in respect to IDA alone.

Next, we performed Fourier-transform infrared spectroscopy (FTIR) to further evaluate the interactions between PtNPs and IDA (Figure 4). The analysis revealed spectral changes in regions associated with carbonyl- and CH-related vibrations, which are consistent with alterations in the local molecular environment of IDA in the presence of PtNPs. However, these assignments should be considered tentative, as FTIR alone does not allow unequivocal identification of the interaction sites. In all analysed spectra, weak absorption features were observed in the 2500–2600 cm^−1^ region. These bands have been previously attributed to CH_n_ groups [49] or to hydrogen-linked bond stretching of alcohols, phenols, carbohydrates, and carboxylic acids. They are commonly interpreted as overtone and combination bands involving deformation modes of CH_3_ and CH_2_ groups located in the 1400–1300 cm^−1^ range (1402 and 1371 cm^−1^ for nanosystems, and 1402 and 1375 cm^−1^ for free amorphous IDA). Although such weak features are present in many hydrocarbons, their precise energy positions are strongly influenced by the local bonding environment. The observed spectral changes are consistent with the possibility that hydrophobic interactions contribute to the association between IDA and PtNP surfaces. Furthermore, additional bands were detected at 2707, 2663, 2611 and 2536 cm^−1^, whereas in the amorphous IDA only a band at 2542 cm^−1^ was registered. The band near 2536 cm^−1^ may be associated with vibrational contributions from CH-containing groups within the anthraquinone-containing anthracycline structure. The feature at 2707 cm^−1^ in the crystalline IDA may be associated with CH_3_ overtone or combination bands, similar to those observed at 2790, 2663 and 2611 cm^−1^. Therefore, the reduction in band intensity in the amorphous state of IDA, accompanied by a shift from 2542 to 2546 cm^−1^ in the samples with PtNPs, may reflect changes in the local vibrational environment of the substituted anthraquinone system and is consistent with possible intermolecular interactions between IDA and PtNPs [50].

The absorption band corresponding to the C=O stretching vibration of IDA was observed in the 1750–1550 cm^−1^ region. To enhance band resolution, a Savitzky–Golay second-derivative treatment was applied, as the use of second derivatives of the spectra can be a fruitful approach for the purpose of revealing unresolved bands, particularly in the context of complex spectra [51,52]. The intensity variations and subtle band shifts in the 2600–2500 cm^−1^ and 1750–1550 cm^−1^ regions align well with the DLS and AFM findings. The formation of PtNP-IDA heteroaggregates and PtNP homoaggregates modifies the local intermolecular packing and hydrogen-bonding networks, which is reflected in altered vibrational profiles of the combined systems. The processed spectra revealed differences in the positions and relative intensities of several vibrational components identified at 1714, 1622, and 1586 cm^−1^. The second-derivative analysis also demonstrated variations in the -C=C- stretching region, with bands observed at 1562 and 1531 cm^−1^, particularly pronounced for samples containing 50 and 70 nm nanoparticles. The observed spectral changes may be related to the absence of steric hindrance from the methoxy (-OCH_3_) substituent in IDA, which could permit closer association of the anthraquinone core with the PtNP surface. Such proximity may alter the local vibrational environment of the aromatic ring, giving rise to the observed spectral changes. Additional alterations were detected near 1493 cm^−1^, where shifts to 1493 and 1483 cm^−1^ were observed, typically associated with aromatic -C=C- vibrations. These spectral changes are consistent with perturbations in the local chemical environment rather than direct evidence for specific binding modes. All registered changes are presented in the Supplementary Information (Table S4). The spectral shifts observed by FTIR, particularly in regions associated with C=O and aromatic vibrations, are consistent with changes in the local molecular environment of IDA. Together with the fluorescence attenuation, these findings support the possibility that IDA molecules associate with PtNPs and may reside in close spatial proximity to the nanoparticle surface.

**Figure 5:**
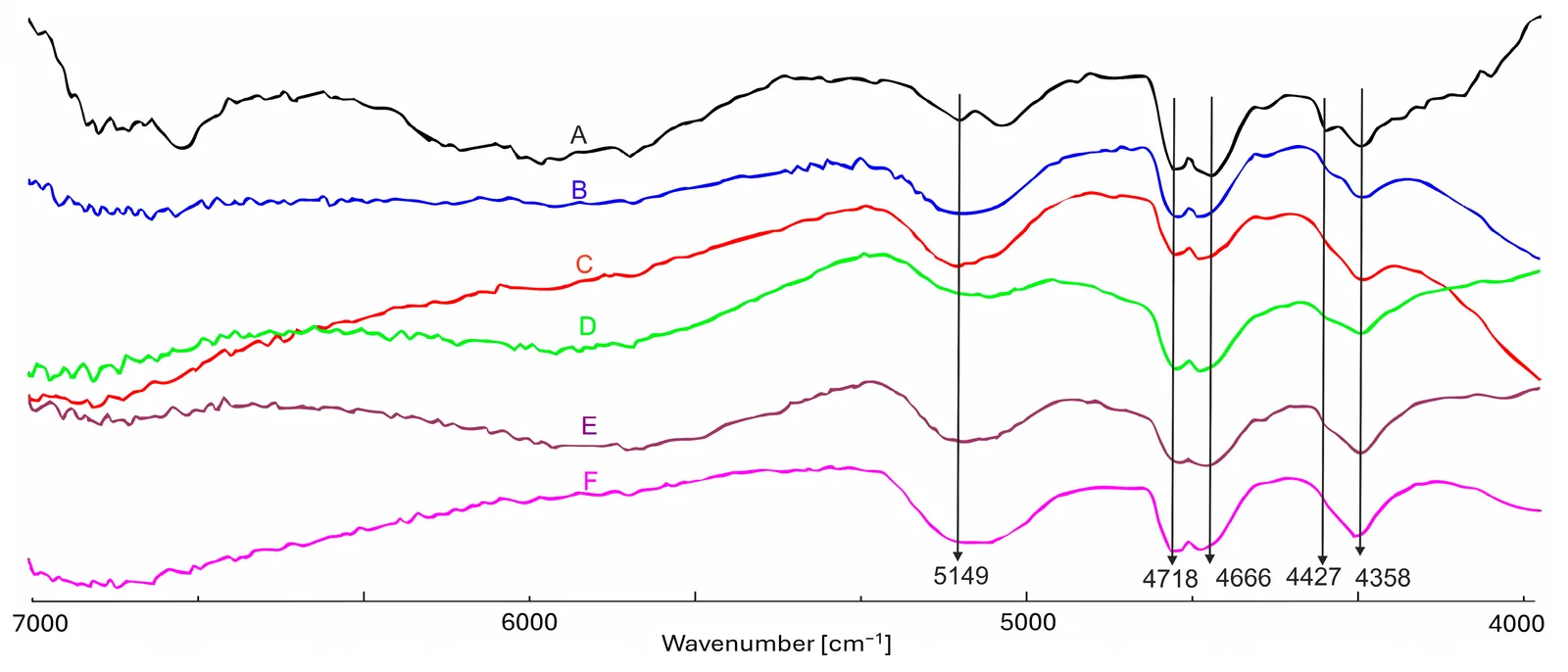
**Near-Infrared Spectroscopy (NIR) spectra of the investigated combinations of idarubicin (IDA) and platinum nanoparticles (PtNPs) in the wavenumber region 7000–4000 cm^−1^.** (A) Black—IDA; (B) blue—IDA lyophilised from sodium citrate; (C) red—5 nm PtNPs with IDA; (D) green—30 nm PtNPs with IDA; (E) violet—50 nm PtNPs with IDA; (F) pink—70 nm PtNPs with IDA.

While FTIR primarily probes fundamental vibrational modes, NIR detects overtone and combination bands that are particularly sensitive to hydrogen bonding and subtle changes in the molecular environment. Therefore, NIR was used as a complementary technique to assess whether the spectral changes observed by FTIR were also reflected in these broader vibrational features (Figure 5). The NIR spectra showed subtle changes in spectral regions associated with O-H, N-H, C-O and C-H overtone and combination bands. These changes were most pronounced for samples containing 50 and 70 nm PtNPs. Another difference was detected in the intensity of the band corresponding to C-O and C-H vibrations (including aromatic C-H and CH_2_ groups), with the highest intensities observed for samples containing 50 and 70 nm PtNPs, particularly at 4358 cm^−1^ and 5149 cm^−1^. The detailed data is available in the Supplementary Information (Table S5).

Overall, the FTIR and NIR results consistently reveal spectral changes that are compatible with possible interactions between IDA and PtNPs and support the presence of changes in the local molecular environment, being in agreement with the aggregation and fluorescence attenuation results. However, these techniques do not enable definitive identification of binding sites or determination of the underling interaction mechanism. Nevertheless, similar spectral trends have been reported previously for PtNP systems involving O-H, N-H and carbonyl-containing compounds [21,40,52,53].

### 2.3. PtNPs-IDA Interactions Are Endothermic

**Figure 6:**
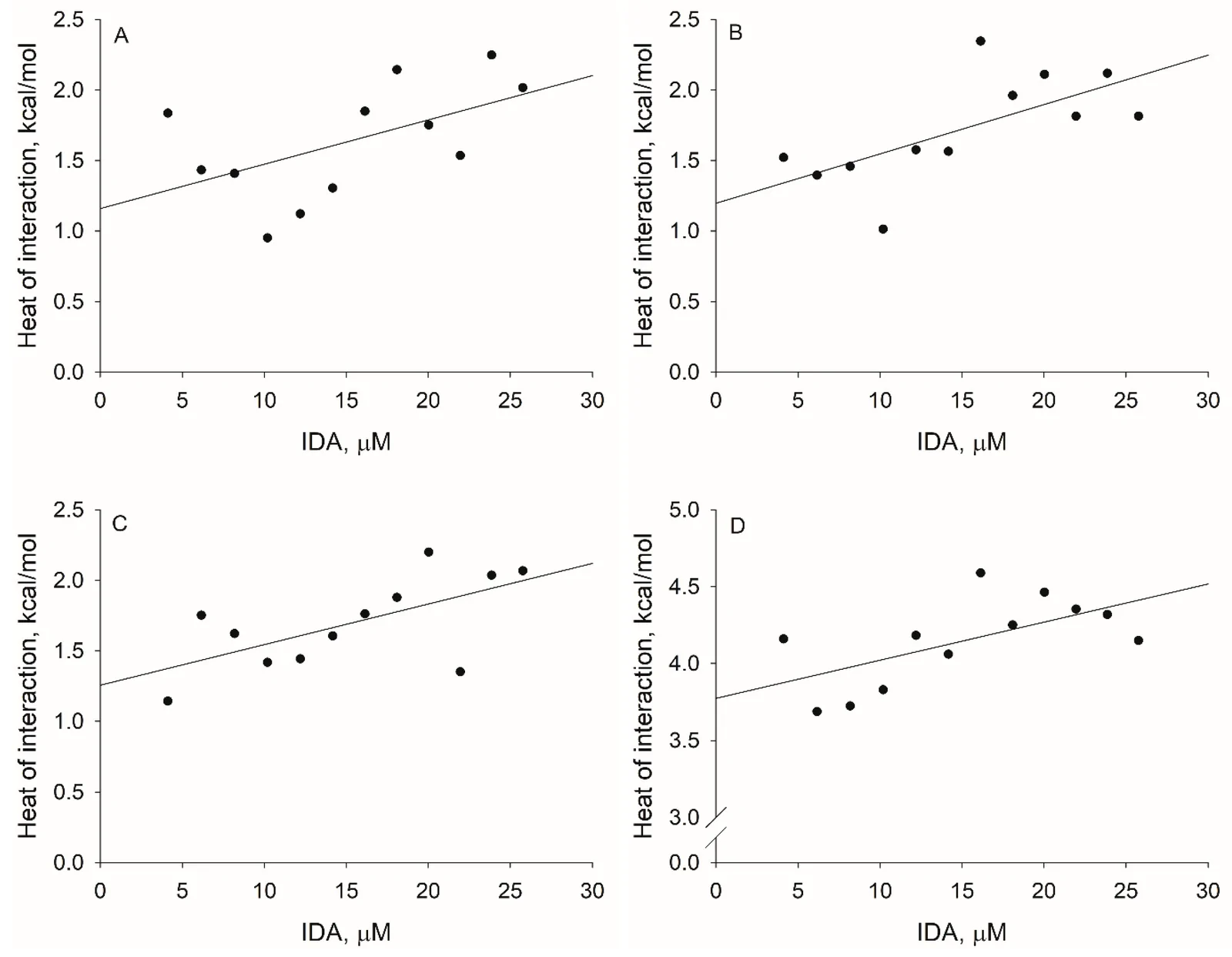
**Isothermal titration calorimetry analysis for platinum nanoparticle (PtNP) interactions with idarubicin (IDA):** (**A**) 5 nm PtNPs (Δ*H* = 1.16 ± 0.25 kcal/mol); (**B**) 30 nm PtNPs (Δ*H* = 1.2 ± 0.21 kcal/mol); (**C**) 50 nm PtNPs (Δ*H* = 1.26 ± 0.18 kcal/mol); (**D**) 70 nm PtNPs (Δ*H* = 3.77 ± 0.1 kcal/mol). IDA concentration range, 2–29 µM; initial PtNPs concentration, 0.05 mg/mL.

In the next step, we investigated the thermodynamic characteristics of the observed PtNP-IDA heteroaggregates. Since the interactions between PtNPs and IDA are very weak, we could not directly determine the complete thermodynamic profile, including binding constants, Gibbs free energy (ΔG), enthalpy (ΔH), and entropy (TΔS) [54]. However, the enthalpy measured directly in an ITC experiment represents the sum of all energetic contributions from the individual equilibria established during the association process. Therefore, the enthalpy changes specific to the interaction between PtNPs and IDA was calculated using an equation based on Hess’s law. The obtained results revealed that the net thermal sign associated with the mixing process is endothermic (Figure 6), with the enthalpy ranging from 1.16 to 3.77 kcal/mol. Interestingly, we did not observe a notable change in the case of nanoparticles sized 5 to 50 nm; however, the 70 nm PtNPs yielded the highest registered total heat. The raw thermal changes attributed to the interactions between PtNPs and IDA are available in the Supplementary Information (Figure S2). The registered positive enthalpy changes indicate that observed heteroaggregation is an endothermic process. From a thermodynamic perspective, spontaneous association requires a negative Gibbs free energy (ΔG < 0). As ΔG = ΔH − TΔS, an observed positive enthalpy (ΔH > 0) is compatible with spontaneous association only if compensated by favourable entropic contribution. Therefore, assuming that the aggregation observed through DLS, AFM, and spectroscopic analyses is associated with a favourable free-energy change, it would necessarily require a compensating entropic contribution. No binding constant, ΔG, or ΔS could be extracted from the ITC data; hence, this interpretation is proposed as a plausible thermodynamic explanation and hypothesis that the process is entropy-driven. In our previous studies, we observed that the enthalpy values increased with the diameter of platinum nanoparticles when combined with other anthracyclines. However, in the case of DOX and EPI, the values registered for 5–50 nm PtNPs were negative, exhibiting an exothermic reaction [37,38]. Furthermore, negative enthalpy values were also observed for PtNPs in combination with acridine mutagen ICR-191 [55], as well as for naked AgNPs [56]. This variability in the thermal behaviour between metallic nanoparticles and different compounds suggests that interactions with specific drugs should be characterised individually, and results obtained for a particular nanoparticle-drug system should not be directly extrapolated to other, even closely related systems.

**Figure 7:**
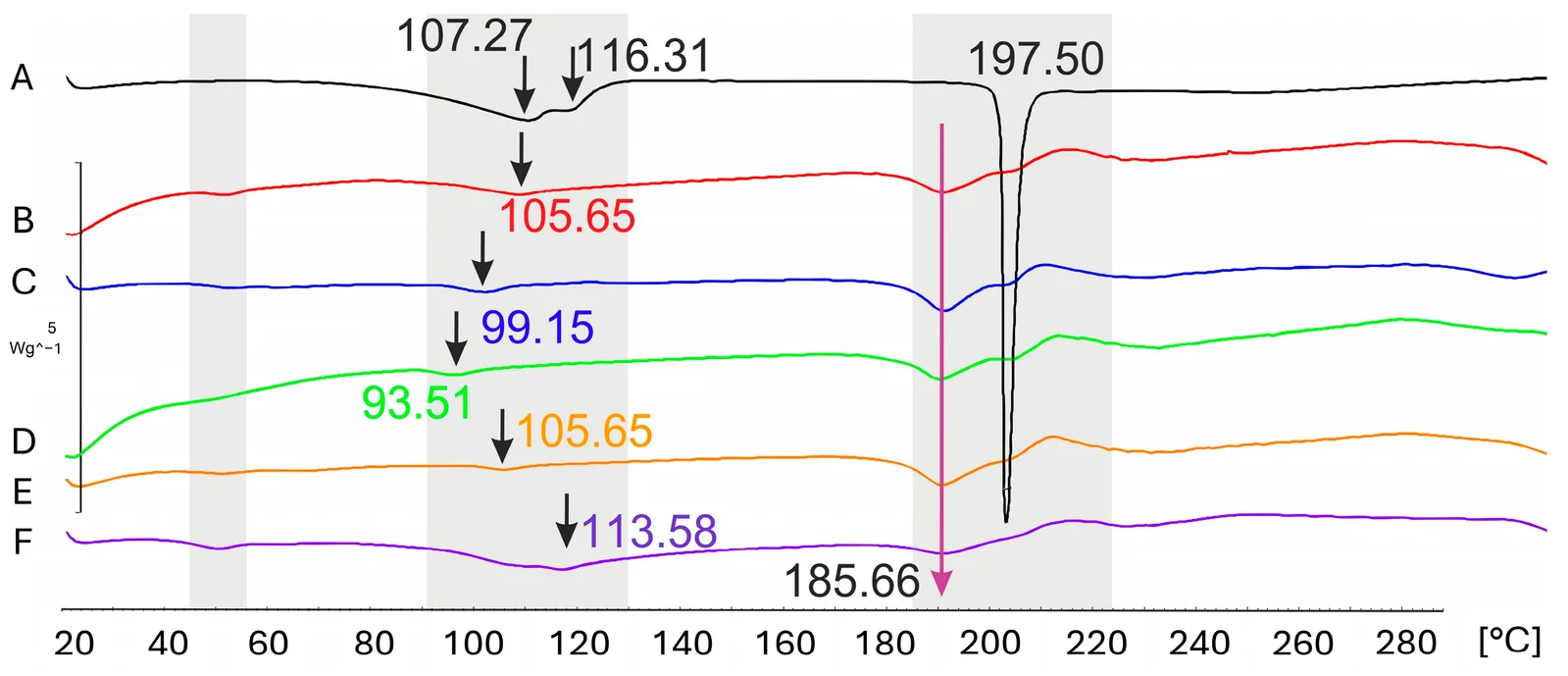
**Differential scanning calorimetry (DSC) curves of platinum nanoparticles (PtNPs) and idarubicin (IDA).** (A) Black—IDA; (B) red—IDA lyophilized from sodium citrate; (C) blue—5 nm PtNPs with IDA; (D) green—30 nm PtNPs with IDA; (E) orange—50 nm PtNPs with IDA; (F) violet—70 nm PtNPs with IDA. Gray areas – regions of significant thermal changes in respect to IDA alone.

Molecular interactions between IDA and PtNPs may influence the chemical structure and association state of the compound, which can manifest as shifts in endothermic and exothermic transitions [57,58]. Therefore, we decided to utilise differential scanning calorimetry (DSC) to evaluate how PtNPs affect the melting transition temperature of IDA (Figure 7). The DSC thermograms revealed that in the range of 93–113 °C, an endothermic peak arises, which can be attributed to moisture loss or possible polymorphic transitions at lower temperatures. The pure IDA is characterised by a melting peak at T = 197 °C. However, when IDA was prepared as a lyophilised sample from sodium citrate solution, this peak shifted to T = 185 °C. When IDA was combined with PtNPs, the melting point shifted significantly only in the case of 70 nm PtNPs, whereas changes for the remaining PtNPs sizes were negligible. The exact data can be found in the Supplementary Information (Table S6).

Overall, the PtNP-IDA systems demonstrated similar thermal behaviour. However, the system containing 70 nm nanoparticles showed transitions at slightly higher temperatures, indicating a more pronounced alteration of the thermal properties compared with IDA alone. A possible explanation for the observed variation in thermal properties could be that with the increase in nanoparticle size from 5 to 70 nm, the surface-area-to-volume ratio decreases by approximately 14 times, which results in a reduced surface curvature and could contribute to stronger or more extended adsorption of IDA onto the nanoparticle surface [59,60].

### 2.4. PtNPs Influence IDA’s Biological Activity

**Figure 8:**
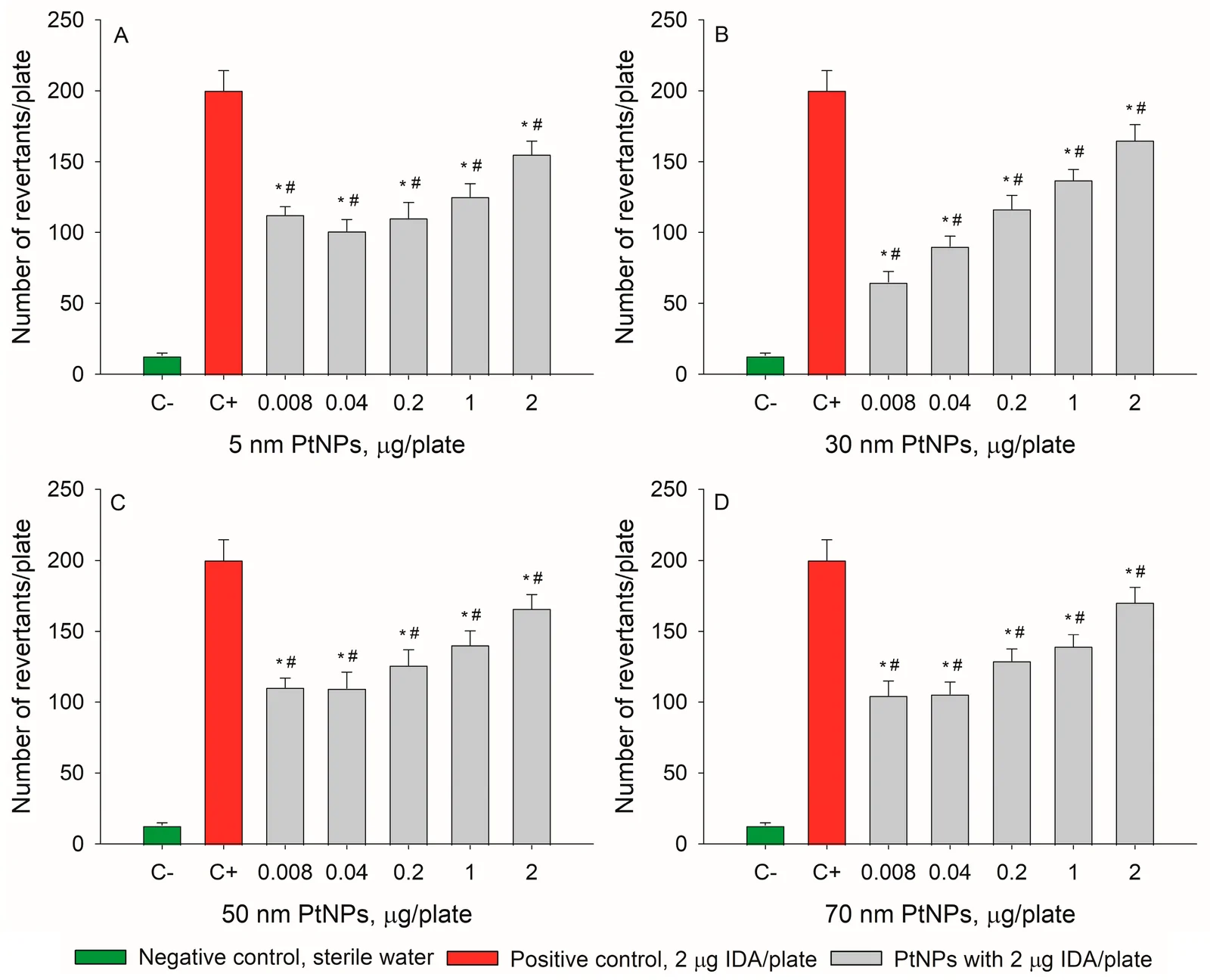
**Influence of platinum nanoparticles (PtNPs) on mutagenicity of idarubicin (IDA) in *Salmonella enterica* serovar Typhimurium TA98 strain:** (**A**) 5 nm PtNPs; (**B**) 30 nm PtNPs; (**C**) 50 nm PtNPs; (**D**) 70 nm PtNPs. C- negative control (sterile water); C+ positive control (2 µg IDA/plate). Results are reported as the average number of revertants per plate ± SD. *—significant difference from the negative control; #—significant difference from the positive control (*p* < α; α = 0.05).

Subsequently, we decided to investigate PtNP-IDA heteroaggregation influence on the biological activity of IDA. For this purpose, the Ames mutagenicity plate assay was selected as a preliminary and straightforward approach to investigate whether PtNPs modify the mutagenic potential of IDA in the *Salmonella enterica* serovar Typhimurium TA98 bacterial model (Figure 8). The observed results show a significant decrease in IDA mutagenicity upon addition of PtNPs of all tested sizes and concentrations, as evidenced by a decrease in the number of revertant colonies relative to the positive control (IDA alone). However, as the concentration of PtNPs increased, the number of revertant colonies gradually returned to the baseline of the positive control, suggesting partial restoration of IDA-induced mutagenicity. Importantly, incubation with PtNPs alone did not increase the number of revertant colonies in the tested bacterial strain, indicating the absence of detectable mutagenic effects of free PtNPs (Figure S3). It has to be noted that the evaluation was conducted on one bacterial strain, however carefully chosen on the basis of literature reports [61], and therefore has to be approached with caution when considering general mutagenic safety.

The observed effect stands in contrast to the results obtained for PtNP interactions with DOX [37], EPI [38] or DAU [39], where increasing PtNP concentration resulted in a gradual decrease in the number of revertant colonies, presumably by reducing the mutagenic capability of the API. This phenomenon can be attributed to numerous mechanisms. Firstly, in contrast to the aforementioned anthracyclines, IDA lacks the methoxy group at the R_1_ position, thus making it the most lipophilic of all these analogues [25]. This feature may promote more efficient membrane penetration by IDA and could contribute to increased intracellular accumulation and ROS generation. Moreover, among the consequences of increased lipophilicity is an increase in the affinity towards hydrophobic surfaces, which PtNPs present. Taking this into consideration, we hypothesise that at lower concentrations of PtNPs, the API-to-nanoparticle ratio is high. As a result, a larger fraction of the PtNP surface may become associated with IDA molecules, which reduces the drug’s bioavailability and leads to a lower number of revertant colonies. However, with a rise in PtNP concentration, the total surface area increases, accompanied by a lower API-to-nanoparticle ratio, which may shift the adsorption–desorption equilibrium [62]. Furthermore, higher concentrations of PtNPs may result in an increased homoaggregation of the nanoparticles, as well as changes in the relative contribution of PtNP homoaggregation and PtNP-IDA heteroaggregation. These processes could increase the fraction of free, biologically active IDA, explaining the partial recovery of mutagenic activity observed at intermediate PtNP concentrations. Additionally, at the highest PtNP concentrations, extensive aggregation may reduce nanoparticle availability to bacterial cells and consequently limit their protective effects. Nevertheless, a fraction of IDA may remain associated with or entrapped within PtNP aggregates, which could explain why its mutagenic activity remains below that of the positive control even at the highest PtNP concentration. Although these mechanisms remain hypothetical, the obtained results demonstrated that PtNPs can influence the biological activity of IDA in a bacterial model, significantly reducing the revertant colonies, presumably by altering the mutagenic potential of IDA in a dose-dependent manner. Based on these observations, we subsequently evaluated their influence in a eukaryotic system.

**Figure 9:**
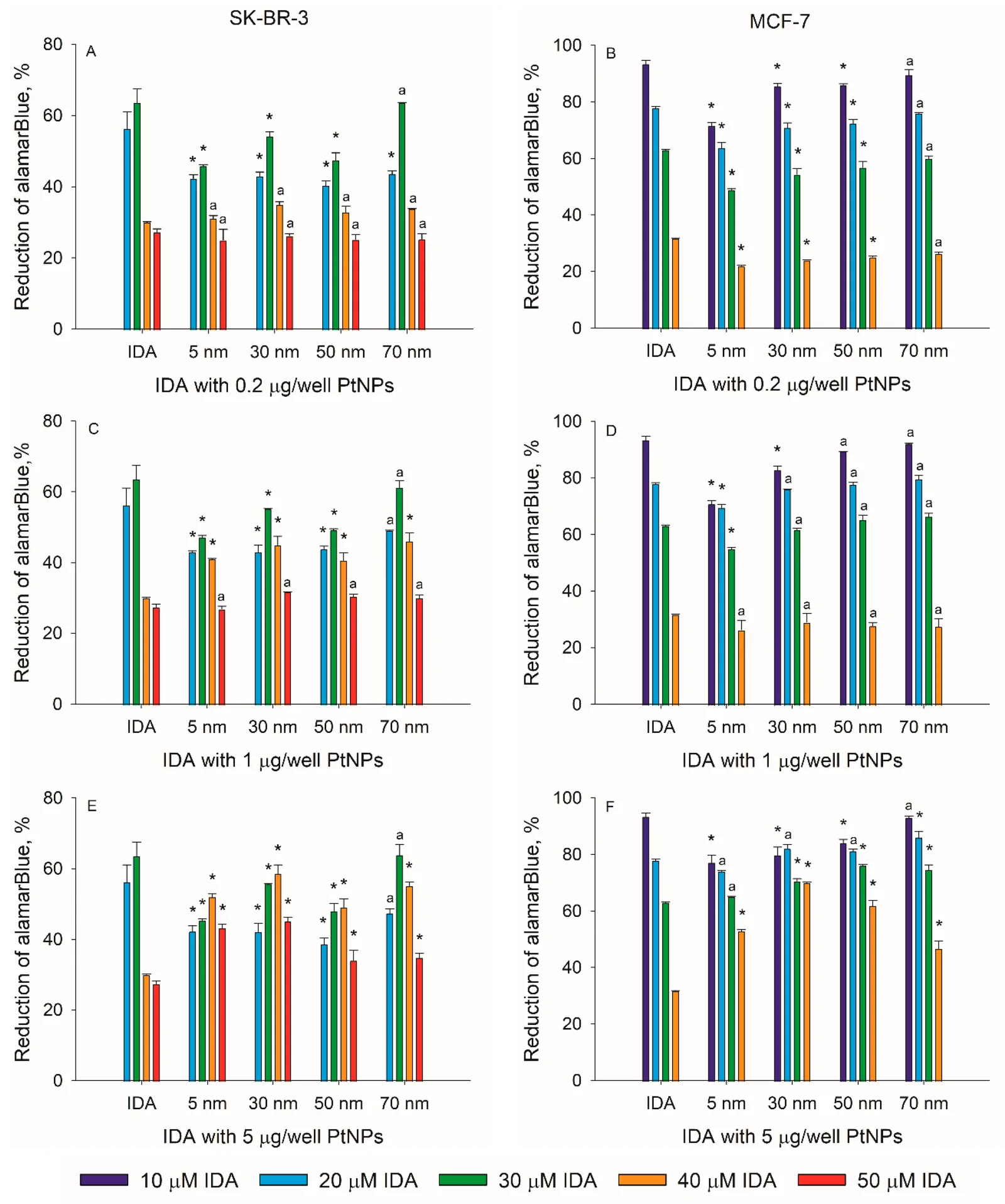
**Influence of platinum nanoparticles (PtNPs) on the cytotoxicity of idarubicin (IDA) on SK-BR-3 and MCF-7 cell lines.** (**A**,**C**,**E**) Influence of 5, 30, 50, and 70 nm PtNPs on IDA’s cytotoxicity at 0.2, 1 and 5 µg/well on the SK-BR-3 cell line; (**B**,**D**,**F**) influence of 5, 30, 50, and 70 nm PtNPs on IDA’s cytotoxicity at 0.2, 1 and 5 µg/well on the MCF-7 cell line. Results are reported as the mean reduction of alamarBlue in regard to the cells treated with the same concentration of IDA alone ± standard deviation (SD). a—no significant difference; *—significant difference (*p* < α, α = 0.05).

Although IDA is predominantly used in the treatment of leukaemia, anthracyclines are also employed in the treatment of solid tumours. Therefore, we investigated whether combining IDA with PtNPs could potentially contribute to repurposing this drug for applications beyond haematological malignancies. For that reason, we performed our analyses on two breast cancer cell lines, SK-BR-3 and MCF-7, differing in receptor expression profiles, with SK-BR-3 being more aggressive (HER2+) [63] (Figure 9). In SK-BR-3, at IDA concentrations of 20 and 30 µM, the combination with most of the PtNP sizes was associated with a reduction in cell viability compared to IDA alone, irrespective of the administered PtNP concentration. However, at 40 and 50 µM IDA, we observed either no significant effects or a decrease in the PtNP-IDA toxicity towards the cells. In MCF-7 cells, the addition of 0.2 µg/well of PtNPs altered the response to all tested IDA concentrations, except for 70 nm PtNPs (Figure 9B). At 1 µg/well, these effects were less pronounced, and with 5 µg/well, we observed similar effects as those for SK-BR-3; with the increase in IDA concentration, the viability of the cells increased (Figure 9D,F). Across both SK-BR-3 and MCF-7 cell lines, 5 nm PtNPs most consistently produced statistically significant changes relative to IDA alone, whereas the effects of larger nanoparticles, particularly 70 nm, were less consistent and were frequently not significantly different from IDA treatment. Almost all PtNPs exhibited anticancer activity of their own towards both cell lines in a dose-dependent manner (Figure S4). Importantly, PtNPs alone did not significantly affect the alamarBlue readouts at either of the examined concentrations, as evidenced by a cell-free interference assay (Figure S5).

The findings suggest that the interaction between PtNPs and IDA is strongly dependent on the concentrations of both components. At lower concentrations, PtNP-IDA combinations were more cytotoxic than IDA alone, whereas increasing the concentrations of API and/or PtNPs progressively diminished this effect and, particularly at 5 µg/well PtNPs, we observed increased cell viability in contrast to the cells treated with an identical concentration of IDA. Although the efficacy of IDA alone against both selected cell lines has been previously reported [64,65], there is limited data available on the combination of IDA with metallic nanoparticles. In our previous studies on anthracyclines and platinum nanoparticles, we observed that PtNPs, at similar tested sizes and concentrations, exerted a protective effect in the non-cancerous HaCaT cell line when combined with anticancer agents, while simultaneously improving cytotoxicity against the cancerous MelJuSo cell line at the highest concentration of each nanoparticle [37]. In contrast, with daunorubicin, we did not observe major changes in the cytotoxicity of PtNP-DAU combinations [39], yet with epirubicin, we have shown that, at 5 µg/well, all the PtNP-EPI combinations presented increased toxicity towards the MelJuSo cell line, while at the same time exhibiting protective effects on HaCaT cells [38]. Furthermore, other research groups have determined that PtNPs enhance the therapeutic efficacy of DOX in B16F10 melanoma and A549 lung cancer cell lines [21], as well as MCF-7 cell lines [40], in a time- and dose-dependent manner. Interestingly, no significant cytotoxicity was demonstrated towards the non-cancerous HCT116 cells, indicating a degree of selectivity towards malignant cells [40]. Thus, IDA appears to produce a distinct response pattern compared to other anthracyclines, and we acknowledged several factors that may contribute to these differences. Firstly, as mentioned in the Ames test discussion, it is worth recalling that of these anthracyclines, IDA is the most lipophilic; therefore, its interactions with PtNPs may present a different profile than what we have previously observed. Secondly, PtNPs can act as both prooxidant and antioxidant agents [66,67]. When administered alone, they exhibited anticancer activity, and in combination with lower concentrations of IDA, they exerted greater cytotoxicity than IDA alone, possibly through enhanced redox cycling. However, as the concentration of both IDA and PtNPs increased, the effects started to diminish, and the viability of cells gradually increased. The observed reduction in cytotoxicity could be partly explained by increased PtNP aggregation, as demonstrated by DLS and AFM analyses. Consistent with our earlier hypothesis, IDA-induced PtNP homoaggregation may reduce the effective interaction of nanoparticles with cells by generating larger aggregates with lower cellular accessibility. In addition to that, IDA may become associated with these aggregates or entrapped within them, reducing the fraction of free, biologically active drug available to the cells. Together, these processes could contribute to the reduced cytotoxicity observed at higher concentrations of IDA and PtNPs. However, it should be emphasised that the present study did not include nanoparticle uptake, intracellular PtNP quantification, or localisation analyses. Therefore, the proposed mechanism should be regarded as a hypothesis that requires verification in future studies.

The proposed mechanism provides one possible explanation for the seemingly contradictory results between the Ames test and cytotoxicity assays. The complexity of the interaction pattern, dependent on the dosage of the two, shows that the combination of PtNPs and IDA may be of great potential. However, the concentration-dependent interaction between these two components highlights a narrow window in which possible synergistic effects may be achieved, and beyond which antagonistic or protective effects may predominate. The differences between our findings and those obtained with other anthracyclines may also reflect the distinct biological characteristics of the models used. While DOX and EPI were evaluated in HaCaT and MelJuSo cells, the present study employed breast cancer cell lines, which may differ in their mechanisms of damage repair and susceptibility to individual anthracyclines. Notably, the effects observed in our study were generally more pronounced in the case of SK-BR-3 cells. This difference may arise from the distinct cell line characteristics, potentially due to the overexpression of the HER2 receptor in SK-BR-3 cells and associated differences in signalling pathways.

Overall, the results of our study indicate that PtNPs interact with IDA and modify its biological activity, affecting both its mutagenic potential towards chosen bacterial strains and its cytotoxic effects against SK-BR-3 and MCF-7 breast cancer cells. However, an increase in PtNP-IDA cytotoxicity in comparison to IDA alone was observed only under specific conditions, highlighting the need for further investigation of this combination. Importantly, increasing the PtNP concentration did not consistently result in greater cytotoxicity. Instead, higher nanoparticle concentrations could diminish the activity of the combination and, in some cases, increase cell viability. Thus, our findings highlight the importance of carefully optimising the relative concentrations of both components, demonstrating that in nanoparticle-based drug combinations, more is not necessarily better. In particular, studies using healthy cell models are necessary to determine whether PtNP-IDA combinations can provide improved selectivity toward cancer cells. Moreover, further preclinical studies, including in vivo models, will be required to assess the safety, efficacy, and translational potential of this approach.

## 3. Materials and Methods

### 3.1. Materials

Platinum nanoparticles (PtNPs) with concentrations of 0.05 mg/mL and 1 mg/mL were purchased from nanoComposix (San Diego, CA, USA). The nanoparticles were supplied in 2 mM sodium citrate solution for 5, 30 and 50 nm PtNPs and in 4 mM sodium citrate solution for 70 nm PtNPs. Idarubicin hydrochloride (IDA) was purchased from Molnova (Ann Arbor, MI, USA). IDA stock solutions were prepared by dissolving the compound in deionised water, and the final concentrations were confirmed spectrophotometrically using the molar extinction coefficient of ε_526.5_ = 3.64 × 10^3^ M^−1^cm^−1^ [68]. The *Salmonella enterica* serovar Typhimurium TA98 strain, used in the Ames mutagenicity assay, was acquired from Xenometrix (Allschwil, Switzerland). All reagents required for bacterial cultivation and mutagenicity testing, including Nutrient Agar, Nutrient Broth, glucose, Ampicillin, L-Histidine, biotin, and necessary salts, were purchased from Sigma-Aldrich (Saint Louis, MO, USA) and used according to the protocol of the Ames *Salmonella*/microsome mutagenicity assay [69]. The SK-BR-3 and MCF-7 cell lines were provided by the Laboratory of Molecular Enzymology and Oncology, Intercollegiate Faculty of Biotechnology, University of Gdansk and Medical University of Gdansk. Dulbecco’s modified Eagle’s medium (DMEM) and 10% foetal bovine serum (FBS), 4 mM L-glutamine, glucose, and antibiotic-antimycotic solution were purchased from ThermoFisher Scientific (Waltham, MA, USA).

### 3.2. Methods

#### 3.2.1. Dynamic Light Scattering (DLS)

The DLS measurements were performed to determine the hydrodynamic diameter of PtNPs and to assess changes in particle size following the addition of IDA. Analyses were carried out at 25 °C in polystyrene cuvettes using a Zetasizer Nano S instrument (Malvern Panalytical, Malvern, UK). PtNPs were measured at an initial concentration of 2.38 µg/mL, while IDA was at a concentration range of 5–50 µM. All experiments were performed in triplicate, with each repetition comprising six measurements, each 10 s in length. The raw acquisition data and analysis were carried out using Zetasizer software v7.13.

#### 3.2.2. Atomic Force Microscopy (AFM)

AFM characterisation of PtNPs and PtNP-IDA systems was carried out at 23 °C in air using a BioScope Resolve atomic force microscope (Bruker, Bremen, Germany), operating in PeakForce Tapping mode. To prepare the samples, PtNPs at a concentration of 0.05 mg/mL were combined with IDA (0.187 µM) and incubated at room temperature. Freshly cleaned mica substrates were first treated with 100 mM cysteamine for 5 min to ensure the adhesion of the PtNPs, and the solution was aspirated afterwards. The prepared suspensions were then deposited onto the modified mica surface and allowed to adsorb for an additional 5 min. Following the incubation, the substrates were rinsed with deionised water and dried under a stream of nitrogen gas before imaging. AFM images were acquired using a ScanAsyst-Fluid+ probe (Bruker, Bremen, Germany) with a resonant frequency of f0 = 150 kHz, and a spring constant of 0.7 N/m. The images were registered at a resolution of 512 × 512 pixels using a PeakForce Tapping frequency of 1 kHz and an oscillation amplitude of 150 nm. The resulting topographic images were generated from the height sensor signal and processed with NanoScope Analysis software v1.9.

#### 3.2.3. Fluorescence Spectroscopy

The fluorescence measurements were conducted in quartz cuvettes at 25 ± 0.1 °C using a Jasco FP-8500 spectrofluorometer (Jasco, Easton, MD, USA) equipped with a Peltier thermostat. All experiments were performed in 2 mM sodium citrate for 5, 30, and 50 nm PtNPs and in 4 mM sodium citrate for 70 nm PtNPs. Each sample contained an initial IDA concentration of 37.2 µM and was subsequently titrated with PtNPs to achieve nanoparticle concentrations between 0 and 10.62 µg/mL. Control samples received equivalent volumes of the corresponding sodium citrate solution instead of PtNPs.

#### 3.2.4. Fourier-Transform Infrared Spectroscopy (FTIR) and Near-Infrared Spectroscopy (NIR)

FTIR and NIR spectra of the associations formed between IDA and PtNPs were recorded using Jasco-4700 spectrometer (Jasco Corporation, Tokyo, Japan). The experiments were conducted in the wavelength range of 4000–400 cm^−1^ for the IR and 8200–4000 cm^−1^ for the NIR, with 32 scans and 4 cm^−1^ resolution. The samples were lyophilised prior to the procedure. NIR transmission spectroscopy was carried out using lyophilised samples of IDA, PtNPs, and sodium citrate. Prior to analysis, each sample was mixed with potassium bromide (KBr) and compressed into a pellet. Background spectra were acquired before sample measurements, and the resulting spectra were analysed using Spectra Analysis software v2.13.00 (Jasco Corporation, Tokyo, Japan).

#### 3.2.5. Isothermal Titration Calorimetry (ITC)

The ITC experiments were carried out at 25 °C on an AutoITC isothermal titration calorimeter (MicroCal Inc. GE Healthcare, Northampton, USA). Both the sample and reference cells had a volume of 1.4491 mL. Each experiment consisted of 12 sequential injections of IDA solution (10.02 μL per injection), with the first injection being only 2 μL to ensure baseline stabilisation and minimise the effect of diffusion from the syringe tip. IDA concentrations ranged from 2 to 29 µM and were titrated into the sample cell containing PtNPs at an initial concentration of 0.05 mg/mL. The control titrations were performed by injecting IDA into the corresponding sodium citrate solution and sodium citrate solution into PtNPs. The heat contributions from these background measurements were subtracted from the experimental data to correct for dilution effects. Each injection lasted 20 s, with an interval of 210 s between consecutive injections. During all measurements, a continuous stirring at 300 rpm was applied to ensure efficient mixing and homogeneity of the sample solution.

#### 3.2.6. Differential Scanning Calorimetry (DSC)

The thermal properties of the samples were evaluated by DSC using a STAR-1 System (Mettler Toledo, Greifensee, Switzerland), which was calibrated with indium. The measurements were performed in 40 µL aluminium crucibles in a temperature range from 20 °C to 300 °C. To perform the experiments, sample masses of 1–5 mg were carefully and accurately weighed. The obtained thermograms were recorded and processed using STAR-1 software v18.30 coupled with an intercooler system from Mettler Toledo. The samples were heated at a constant rate of 10 °C/min under a nitrogen atmosphere with a flow rate of 60 mL/min. All measurements were normalised according to the mass of the analysed sample.

#### 3.2.7. Ames Mutagenicity Assay

The influence of PtNPs on the mutagenic activity of IDA was assessed employing Ames mutagenicity plate assay on *Salmonella enterica* serovar Typhimurium TA98 strain, based on the altered OECD Test Guideline No. 471, Bacterial Reverse Mutation Test, described by Woziwodzka et al. [70]. A sample containing 100 µL of overnight bacterial culture, 50 µL of 3% NaCl, and 100 µL of tested solution (or sterile distilled water for the negative control) was incubated for 4 h in darkness at 37 °C and 220 rpm. Subsequently, the sample was centrifuged at 11,840 *g*, and the bacterial pellet was washed with 0.75% NaCl and resuspended in 300 µL of 0.75% NaCl solution with the addition of 0.1 µM histidine and 0.1 µM biotin. Next, the bacterial suspension was swabbed onto a glucose minimal agar plate and incubated in darkness at 37 °C for 48 h. All samples were analysed in three technical replicates. After the incubation, the number of revertant colonies on each plate was counted, and the mean number of colonies, alongside the standard deviation and the statistical significance, was calculated.

#### 3.2.8. Cell Culture and Cytotoxicity Assay

The SK-BR-3 and MCF-7 cell lines were cultivated in Dulbecco’s modified Eagle medium (DMEM) supplemented with 10% foetal bovine serum (FBS), 4 mM L-glutamine, 100 U/mL penicillin, 100 µg/mL streptomycin, and 4500 mg/L glucose. Cells were maintained under standard culture conditions in a humidified incubator at 37 °C with a 5% CO_2_ atmosphere.

For the evaluation of cell viability using the alamarBlue reduction assay, SK-BR-3 and MCF-7 cells were seeded into 96-well plates at densities of 2 × 10^4^ cells/well and 4 × 10^4^ cells/well, respectively, and allowed to attach overnight under the same incubation conditions. The following day, cells were washed three times with an FBS-free DMEM. Subsequently, cells were treated with PtNPs alone (0.2, 1, and 5 µg/well) or PtNPs combined with IDA at concentration ranges of 20–50 µM for SK-BR-3 cells and 10–40 µM for MCF-7 cells. The above-mentioned treatments were added in a final volume of 90 µL per well and performed in three technical replicates for each sample. Next, the cells were incubated for 20 h at 37 °C in a humidified 5% CO_2_ atmosphere. Following this incubation, 10 µL of alamarBlue reagent (BioRad) was added to each well, and the plates were incubated for an additional 4 h under identical conditions. Finally, the absorbance of each well was measured at 570 nm and 600 nm, and the percentage of alamarBlue reduction was calculated by comparing the treated samples with untreated controls according to the manufacturer’s protocol described below:

$$diff\left(\%\right)=\frac{\left(O_{2}\times A_{1}\right)-(O_{1}\times A_{2})}{\left(O_{2}\times P_{1}\right)-(O_{1}\times P_{2})}$$

where diff is the difference between treated and control cells; O_1_ is the ε_570_ of oxidized alamarBlue (80,586 M^−1^cm^−1^); O_2_ is the ε_600_ of oxidized alamarBlue (117,216 M^−1^cm^−1^); A_1_ is the absorbance of the test wells at 570 nm; A_2_ is the absorbance of test wells at 600 nm; P_1_ is the absorbance of control wells at 570 nm; and P_2_ is the absorbance of control wells at 600 nm.

To account for the possible interaction, an interference assay was performed. Mixtures of PtNPs alone at the above-mentioned concentrations were prepared in a cell-free and FBS-free medium. Next, the samples were added in a final volume of 90 µL per well and performed in three technical replicates, and incubated for 20 h at 37 °C in a humidified 5% atmosphere. Following this incubation, 10 µL of alamarBlue reagent (BioRad) was added to each well, and the plates were incubated for an additional 4 h under identical conditions. Finally, the absorbance of each well was measured and calculated according to the protocol described above.

#### 3.2.9. Computer Analysis

Data visualisation was performed using SigmaPlot v.14.5 from Systat Software Inc. (San Jose, CA, USA). The statistical analyses were carried out in Statistica 14.0 from TIBCO Software Inc. (Palo Alto, CA, USA). Statistical differences in the Ames mutagenicity and cytotoxicity assays were assessed by one-way ANOVA followed by Tukey’s HSD multiple-comparison test. Differences were considered statistically significant when the *p* value was below α = 0.05.

## 4. Conclusions

In summary, platinum nanoparticles (PtNPs) of distinct sizes (5, 30, 50, and 70 nm) were shown to interact with idarubicin (IDA). The formation of PtNP-IDA heteroaggregates was observed, providing significant insight into the close molecular interactions and suggesting the involvement of amine groups in the formation of these associations. Furthermore, the results indicated that the interactions were mainly endothermic, with the most pronounced changes observed for 70 nm PtNPs. Combinations of PtNP-IDA yielded reduced revertant colonies in comparison to IDA alone, suggesting significant influence of PtNPs on the mutagenic potential of IDA in the chosen bacterial strain. Cytotoxicity analyses performed on the MCF-7 and SK-BR-3 cancer cell lines revealed that PtNPs alone exhibited significant cytotoxicity. However, PtNP-IDA combinations exhibited greater cytotoxicity than IDA alone primarily at lower API concentrations, whereas increasing IDA and PtNP concentrations resulted in a gradual increase in cell viability. In our study, 5 nm PtNPs most consistently exhibited statistically significant changes relative to IDA alone, whereas the largest PtNPs, 70 nm, showed fewer statistically significant differences from the IDA treatment. Overall, these findings suggest that the size of PtNPs and the relative doses of both these components may be important factors in modulating the biological activity of IDA. These observations may provide useful guidance for the rational design and optimization of platinum-nanoparticle-based combination nanomedicine systems, highlighting the possibility of enhanced efficacy of such combinations. However, further research is required, as this study was limited to two cancer cell lines, without a non-cancerous cell line. Furthermore, more mechanistic assays are needed to accurately determine the underlying mechanisms of action, the optimal dose ratio of PtNPs to IDA, and cellular localization of PtNP-IDA combination. Additionally, further research involving a broader range of in vitro models, including leukaemia cell models, as well as subsequent in vivo studies, is required to better elucidate the effects of PtNPs on IDA cytotoxicity and to assess the potential therapeutic relevance of these interactions.

## Data Availability

The original contributions presented in this study are included in the article/Supplementary Material. Further inquiries can be directed to the corresponding author.

## Supplementary Materials

The following supporting information can be downloaded at: https://www.mdpi.com/article/10.3390/ph19081274/s1, Table S1. Characteristics of platinum nanoparticles (PtNPs) provided by the manufacturer. Table S2. Data for DLS analysis of platinum nanoparticles (PtNPs) alone and in combination with idarubicin (IDA). SD—standard deviation; PdI—polydispersity index. Table S3. Data for the AFM analysis of platinum nanoparticles (PtNPs) alone and in combination with idarubicin (IDA). SD—standard deviation. Table S4. Fourier-transform infrared spectroscopic (FTIR) characterisation of idarubicin (IDA) alone and in combination with various sizes of platinum nanoparticles (PtNPs). The regions of significant changes in IDA’s bands are marked in red. Table S5. Near-infrared spectroscopic (NIR) characterisation of idarubicin (IDA) and its complexes with various sizes of platinum nanoparticles (PtNPs). The regions of significant changes in IDA’s bands are marked in red. Table S6. DSC data for idarubicin (IDA) samples combined with platinum nanoparticles (PtNPs) of different sizes. Numbers marked in red indicate significant changes with respect to lyophilised IDA. Figure S1. Changes in the fluorescence of idarubicin (IDA) upon titration with various sizes of platinum nanoparticles (PtNPs) or appropriate sodium citrate solution (buffer): (A,B) 2 mM or 4 mM sodium citrate, respectively; (C–F) 5, 30, 50 and 70 nm PtNPs, respectively. Red line—IDA alone; blue—IDA with the highest titrated volume of PtNPs or buffer. IDA concentration range: 37.2–29.3 µM; PtNPs concentration range: 0–10.62 µg/mL. Figure S2. Thermograms showing microcalorimetric titrations of platinum nanoparticles (PtNPs) with idarubicin (IDA): (A) 5 nm PtNPs; (B) 30 nm PtNPs; (C) 50 nm PtNPs; (D) 70 nm PtNPs. Black—PtNPs with IDA; red—water with PtNPs; cyan—water with appropriate sodium citrate solution; green—IDA with appropriate sodium citrate solution. Figure S3. Mutagenicity of platinum nanoparticles (PtNPs) in *Salmonella enterica* serovar Typhimurium TA98 strain: (A–D) 5, 30, 50 and 70 nm PtNPs, respectively. C- negative control (sterile water); C+ positive control (2 µg IDA/plate). Results are reported as the average number of revertants per plate ± SD. #—significant difference from the positive control (p < α; α = 0.05). Figure S4. Anticancer activity of platinum nanoparticles (PtNPs) on SK-BR-3 and MCF-7 cell lines. Results are reported as the mean reduction of alamarBlue in regard to the untreated cells (negative control, 100% viability) ± standard deviation (SD). a—no significant difference; *—significant difference (p < α, α = 0.05).

## References

[1] Bray F., Laversanne M., Sung H., Ferlay J., Siegel R.L., Soerjomataram I., Jemal A. (2024). Global cancer statistics 2022: GLOBOCAN estimates of incidence and mortality worldwide for 36 cancers in 185 countries. CA Cancer J. Clin..

[2] Siegel R.L., Kratzer T.B., Giaquinto A.N., Sung H., Jemal A. (2025). Cancer statistics, 2025. CA Cancer J. Clin..

[3] Anand U., Dey A., Chandel A.K.S., Sanyal R., Mishra A., Pandey D.K., De Falco V., Upadhyay A., Kandimalla R., Chaudhary A. (2022). Cancer chemotherapy and beyond: Current status, drug candidates, associated risks and progress in targeted therapeutics. Genes Dis..

[4] Sonkin D., Thomas A., Teicher B.A. (2024). Cancer treatments: Past, present, and future. Cancer Genet..

[5] Liu B., Zhou H., Tan L., Siu K.T.H., Guan X.Y. (2024). Exploring treatment options in cancer: Tumor treatment strategies. Signal Transduct. Target. Ther..

[6] Fan D., Cao Y., Cao M., Wang Y., Cao Y., Gong T. (2023). Nanomedicine in cancer therapy. Signal Transduct. Target. Ther..

[7] Soares S., Sousa J., Pais A., Vitorino C. (2018). Nanomedicine: Principles, Properties, and Regulatory Issues. Front. Chem..

[8] Altammar K.A. (2023). A review on nanoparticles: Characteristics, synthesis, applications, and challenges. Front. Microbiol..

[9] Kumari S., Raturi S., Kulshrestha S., Chauhan K., Dhingra S., András K., Thu K., Khargotra R., Singh T. (2023). A comprehensive review on various techniques used for synthesizing nanoparticles. J. Mater. Res. Technol..

[10] Eker F., Duman H., Akdaşçi E., Bolat E., Sarıtaş S., Karav S., Witkowska A.M. (2024). A Comprehensive Review of Nanoparticles: From Classification to Application and Toxicity. Molecules.

[11] Jeyaraj M., Gurunathan S., Qasim M., Kang M.H., Kim J.H. (2019). A Comprehensive Review on the Synthesis, Characterization, and Biomedical Application of Platinum Nanoparticles. Nanomaterials.

[12] Gurunathan S., Jeyaraj M., La H., Yoo H., Choi Y., Do J.T., Park C., Kim J.-H., Hong K. (2020). Anisotropic Platinum Nanoparticle-Induced Cytotoxicity, Apoptosis, Inflammatory Response, and Transcriptomic and Molecular Pathways in Human Acute Monocytic Leukemia Cells. Int. J. Mol. Sci..

[13] Dobrucka R., Romaniuk-Drapała A., Kaczmarek M. (2019). Evaluation of biological synthesized platinum nanoparticles using *Ononidis radix* extract on the cell lung carcinoma A549. Biomed. Microdevices.

[14] Yang C., Wang M., Zhou J., Chi Q. (2017). Bio-synthesis of peppermint leaf extract polyphenols capped nano-platinum and their in-vitro cytotoxicity towards colon cancer cell lines (HCT 116). Mater. Sci. Eng. C.

[15] Bendale Y., Bendale V., Paul S. (2017). Evaluation of cytotoxic activity of platinum nanoparticles against normal and cancer cells and its anticancer potential through induction of apoptosis. Integr. Med. Res..

[16] Waliaveettil F.A., Jose J., Anila E.I. (2024). Chitosan stabilized platinum nanoparticles: Synthesis, characterization and cytotoxic impacts on human breast cancer cells. Mater. Chem. Phys..

[17] Shiny P.J., Mukherjee A., Chandrasekaran N. (2016). DNA damage and mitochondria-mediated apoptosis of A549 lung carcinoma cells induced by biosynthesised silver and platinum nanoparticles. RSC Adv..

[18] Vagena I.A., Malapani C., Gatou M.A., Lagopati N., Pavlatou E.A. (2025). Enhancement of EPR Effect for Passive Tumor Targeting: Current Status and Future Perspectives. Appl. Sci..

[19] Mukherjee S., Kotcherlakota R., Haque S., Bhattacharya D., Kumar J.M., Chakravarty S., Patra C.R. (2020). Improved delivery of doxorubicin using rationally designed PEGylated platinum nanoparticles for the treatment of melanoma. Mater. Sci. Eng. C.

[20] Fu B., Dang M., Tao J., Li Y., Tang Y. (2020). Mesoporous platinum nanoparticle-based nanoplatforms for combined chemo-photothermal breast cancer therapy. J. Colloid Interface Sci..

[21] Faderin E., Iorkula T.H., Aworinde O.R., Awoyemi R.F., Awoyemi C.T., Acheampong E., Chukwu J.U., Agyemang P., Onaiwu G.E., Ifijen I.H. (2025). Platinum nanoparticles in cancer therapy: Chemotherapeutic enhancement and ROS generation. Med. Oncol..

[22] Gurunathan S., Jeyaraj M., Kang M.H., Kim J.H. (2019). Tangeretin-Assisted Platinum Nanoparticles Enhance the Apoptotic Properties of Doxorubicin: Combination Therapy for Osteosarcoma Treatment. Nanomaterials.

[23] Pedone D., Moglianetti M., De Luca E., Bardi G., Pompa P.P. (2017). Platinum nanoparticles in nanobiomedicine. Chem. Soc. Rev..

[24] Touzani A., Ramos-Pan L., Fraga S., Fernández-Bertólez N., Laffon B., Valdiglesias V. (2025). Systematic review on toxicological effects of platinum nanoparticles: Towards their use as safe biomedical tools. Mutat. Res. Rev. Mutat. Res..

[25] Ma P., Mumper R.J. (2013). Anthracycline Nano-Delivery Systems to Overcome Multiple Drug Resistance: A Comprehensive Review. Nano Today.

[26] Ozluer C., Kara H.E.S. (2014). In vitro DNA binding studies of anticancer drug idarubicin using spectroscopic techniques. J. Photochem. Photobiol. B.

[27] Fields S.M., Koeller J.M. (1991). Idarubicin: A Second-Generation Anthracycline. DICP.

[28] Liu F.T., Kelsey S.M., Newland A.C., Jia L. (2001). Generation of reactive oxygen species is not involved in idarubicin-induced apoptosis in human leukaemic cells. Br. J. Haematol..

[29] Bano N., Parveen S., Saeed M., Siddiqui S., Abohassan M., Mir S.S. (2024). Drug Repurposing of Selected Antibiotics: An Emerging Approach in Cancer Drug Discovery. ACS Omega.

[30] Wang H., Xiao X., Xiao Q., Lu Y., Wu Y. (2020). The efficacy and safety of daunorubicin versus idarubicin combined with cytarabine for induction therapy in acute myeloid leukemia: A meta-analysis of randomized clinical trials. Medicine.

[31] Rayner D.M., Cutts S.M. (2023). Anthracyclines. Side Eff. Drugs Annu..

[32] Eker F., Akdaşçi E., Duman H., Bechelany M., Karav S. (2024). Gold Nanoparticles in Nanomedicine: Unique Properties and Therapeutic Potential. Nanomaterials.

[33] Gomes H.I.O., Martins C.S.M., Prior J.A.V. (2021). Silver Nanoparticles as Carriers of Anticancer Drugs for Efficient Target Treatment of Cancer Cells. Nanomaterials.

[34] Shestovskaya M.V., Luss A.L., Bezborodova O.A., Makarov V.V., Keskinov A.A. (2023). Iron Oxide Nanoparticles in Cancer Treatment: Cell Responses and the Potency to Improve Radiosensitivity. Pharmaceutics.

[35] Shahalaei M., Azad A.K., Sulaiman W.M.A.W., Derakhshani A., Mofakham E.B., Mallandrich M., Kumarasamy V., Subramaniyan V. (2024). A review of metallic nanoparticles: Present issues and prospects focused on the preparation methods, characterization techniques, and their theranostic applications. Front. Chem..

[36] Burlec A.F., Corciova A., Boev M., Batir-Marin D., Mircea C., Cioanca O., Danila G., Danila M., Bucur A.F., Hancianu M. (2023). Current Overview of Metal Nanoparticles’ Synthesis, Characterization, and Biomedical Applications, with a Focus on Silver and Gold Nanoparticles. Pharmaceuticals.

[37] Bełdzińska P., Zakrzewski M., Mruk I., Bogusławski M., Derewońko N., Bury K., Wyrzykowski D., Gołuński G., Rychłowski M., Piosik J. (2025). Size dependent impact of platinum nanoparticles on doxorubicin activity. Eur. J. Pharm. Sci..

[38] Bełdzińska P., Zakrzewski M., Grzyb K., Hauer A., Jamrógiewicz M., Wyrzykowski D., Bury K., Gołuński G., Piosik J. (2025). Platinum nanoparticles interact with epirubicin in size dependent manner and affect its biological activity. Eur. J. Pharm. Biopharm..

[39] Zakrzewski M., Bełdzińska P., Gajdowska F., Gołuński G., Gackowska K., Strankowska J., Jamrógiewicz M., Wyrzykowski D., Grzyb K., Gutowska-Owsiak D. (2025). The effect of platinum nanoparticles on the physicochemical properties of daunorubicin. RSC Adv..

[40] Kalındemirtaş F.D., Cilasun G.E., Kariper A. (2025). Enhanced therapeutic efficacy of platinum-doxorubicin nanoparticles on colon and breast cancer cell lines. Naunyn-Schmiedeberg’s Arch. Pharmacol..

[41] Jakhmola A., Hornsby T.K., Kashkooli F.M., Kolios M.C., Rod K., Tavakkoli J. (2024). Green synthesis of anti-cancer drug-loaded gold nanoparticles for low-intensity pulsed ultrasound targeted drug release. Drug Deliv. Transl. Res..

[42] Lesaine A., Bonamy D., Rountree C.L., Gauthier G., Impéror-Clerc M., Lazarus V. (2021). Role of particle aggregation in the structure of dried colloidal silica layers. Soft Matter.

[43] Lakowicz J.R., Ray K., Chowdhury M., Szmacinski H., Fu Y., Zhang J., Nowaczyk K. (2008). Plasmon-controlled fluorescence: A new paradigm in fluorescence spectroscopy. Analyst.

[44] Jeong Y., Kook Y.-M., Lee K., Koh W.-G. (2018). Metal enhanced fluorescence (MEF) for biosensors: General approaches and a review of recent developments. Biosens. Bioelectron..

[45] Patel P., Umapathy D., Manivannan S., Nadar V.M., Venkatesan R., Joseph Arokiyam V.A., Pappu S., Ponnuchamy K. (2021). A doxorubicin–platinum conjugate system: Impacts on PI3K/AKT actuation and apoptosis in breast cancer cells. RSC Adv..

[46] Abd Elhaleem S.M., Elsebaei F., Shalan S., Belal F. (2023). Investigating the Effect of Silver Nanoparticles on the Fluorescence Intensity of Bambuterol and its Active Metabolite Terbutaline Using FRET. J. Fluoresc..

[47] Gołuński G., Konkel K., Galikowska Bogut B., Bełdzińska P., Bury K., Zakrzewski M., Butowska K., Sądej R., Piosik J. (2024). Influence of silver nanoparticles’ size on their direct interactions with doxorubicin and its biological effects. Sci. Rep..

[48] Liu E., Zhang M., Cui H., Gong J., Huang Y., Wang J., Cui Y., Dong W., Sun L., He H. (2018). Tat-functionalized Ag-Fe3O4 nano-composites as tissue-penetrating vehicles for tumor magnetic targeting and drug delivery. Acta Pharm. Sin. B.

[49] Lawson E.E., Edwards H.G.M., Johnson A.F. (1995). FT Raman spectroscopic study of the wavenumber region 2800-2630 cm−1 of selected organic compounds. Spectrochim. Acta Part A Mol. Biomol. Spectrosc..

[50] Duley W.W., Hu A. (2012). The 217.5 nm band, infrared absorption, and infrared emission features in hydrogenated amorphous carbon nanoparticles. Astrophys. J..

[51] Zheng C., Liu H., Li J., Wang Y. (2024). Identification and crude protein prediction of porcini mushrooms via deep learning-assisted FTIR fingerprinting. LWT.

[52] Pasieczna-Patkowska S., Cichy M., Flieger J. (2025). Application of Fourier Transform Infrared (FTIR) Spectroscopy in Characterization of Green Synthesized Nanoparticles. Molecules.

[53] Erdemir-Cilasun G., Özerkan D., Kariper İ.A., Sert E., Korkut I.N., Danışman-Kalındemirtaş F. (2025). Improved apoptosis and mitochondrial dysfunction: The potential of carmofur-platinum nanoparticles. Biomed. Mater..

[54] Turnbull W.B., Daranas A.H. (2003). On the Value of c: Can Low Affinity Systems Be Studied by Isothermal Titration Calorimetry?. J. Am. Chem. Soc..

[55] Borowik A., Banasiuk R., Derewonko N., Rychlowski M., Krychowiak-Masnicka M., Wyrzykowski D., Ziabka M., Woziwodzka A., Krolicka A., Piosik J. (2019). Interactions of newly synthesized platinum nanoparticles with ICR-191 and their potential application. Sci. Rep..

[56] Borowik A., Butowska K., Konkel K., Banasiuk R., Derewonko N., Wyrzykowski D., Davydenko M., Cherepanov V., Styopkin V., Prylutskyy Y. (2019). The Impact of Surface Functionalization on the Biophysical Properties of Silver Nanoparticles. Nanomaterials.

[57] Borges O., Borchard G., Verhoef J.C., De Sousa A., Junginger H.E. (2005). Preparation of coated nanoparticles for a new mucosal vaccine delivery system. Int. J. Pharm..

[58] Wong A.C.Y., Lam F. (2002). Study of selected thermal characteristics of polypropylene/polyethylene binary blends using DSC and TGA. Polym. Test..

[59] Villarreal E., Li G.G., Zhang Q., Fu X., Wang H. (2017). Nanoscale Surface Curvature Effects on Ligand–Nanoparticle Interactions: A Plasmon-Enhanced Spectroscopic Study of Thiolated Ligand Adsorption, Desorption, and Exchange on Gold Nanoparticles. Nano Lett..

[60] Gulumian M., Andraos C., Afantitis A., Puzyn T., Coville N.J. (2021). Importance of Surface Topography in Both Biological Activity and Catalysis of Nanomaterials: Can Catalysis by Design Guide Safe by Design?. Int. J. Mol. Sci..

[61] Babudril N., Panil B., Tamarol M., Monti-Bragadin’ C., Zunino2 F. (1984). Mutagenic and cytotoxic activity of doxorubicin and daunorubicin derivatives on prokaryotic and eukaryotic cells. Br. J. Cancer.

[62] Wang Y., Yang S.T., Wang Y., Liu Y., Wang H. (2012). Adsorption and desorption of doxorubicin on oxidized carbon nanotubes. Colloids Surf. B Biointerfaces.

[63] Azizpour M., Changizzadeh B., Golbashirzadeh M., Moradzadegan A. (2025). Exploring the therapeutic potential of naringin and melatonin in breast cancer: A focus on SKBR3 and MCF-7 cell lines. Biomed. Res. Ther..

[64] Orlandi P., Barbara C., Bocci G., Fioravanti A., Di Paolo A., Del Tacca M., Danesi R. (2005). Idarubicin and Idarubicinol Effects on Breast Cancer Multicellular Spheroids. J. Chemother..

[65] Siavoshinia L., Jamalan M., Zeinali M., Pourshohod A., Koushki M., Moradipoodeh B., Mohammadzadeh G. (2020). Improvement of Targeted Chemotherapy of HER2-positive Ovarian Malignant Cell Line by ZHER2-Idarubicin Conjugate: An in vitro Study. Iran. J. Pathol..

[66] Ismail N.A.S., Lee J.X., Yusof F. (2022). Platinum Nanoparticles: The Potential Antioxidant in the Human Lung Cancer Cells. Antioxidants.

[67] Chen C., Fan S., Li C., Chong Y., Tian X., Zheng J., Fu P.P., Jiang X., Wamer W.G., Yin J.-J. (2016). Platinum nanoparticles inhibit antioxidant effects of vitamin C via ascorbate oxidase-mimetic activity. J. Mater. Chem. B.

[68] Gołuński G., Borowik A., Lipińska A., Romanik M., Derewońko N., Woziwodzka A., Piosik J. (2016). Pentoxifylline affects idarubicin binding to DNA. Bioorg. Chem..

[69] Mortelmans K., Zeiger E. (2000). The Ames Salmonella/microsome mutagenicity assay. Mutat. Res. Fundam. Mol. Mech. Mutagen..

[70] Woziwodzka A., Gwizdek-Wiśniewska A., Piosik J. (2011). Caffeine, pentoxifylline and theophylline form stacking complexes with IQ-type heterocyclic aromatic amines. Bioorg. Chem..

