# Supplementary material for: Platinum Nanoparticles as Modulators of Idarubicin Activity: A Physicochemical and In Vitro Biological Study
Source: Pharmaceuticals (Basel). 2026 Aug 12;19(8):1274. doi: 10.3390/ph19081274 (PMC13517054; doi:10.3390/ph19081274)
Supplement: Supplementary file 1 [file pharmaceuticals-19-01274-s001.zip › pharmaceuticals-4441985-supplementary.pdf]

Table S1. Characteristics of platinum nanoparticles (PtNPs) provided by the manufacturer.

Parameter	5 nm PtNPs	30 nm PtNPs	50 nm PtNPs	70 nm PtNPs
**Hydrodynamic diameter, nm**	Not reported	39	65	78
**Zeta potential, mV**	Not reported	-44	-55	-48
**Platinum purity, %**	99.99	99.99	99.99	99.99
**Particle surface**	Bare (Citrate)	Bare (Citrate)	Bare (Citrate)	Bare (Citrate)

Table S2. Data for DLS analysis of platinum nanoparticles (PtNPs) alone and in combination with idarubicin (IDA). SD – standard deviation, PdI – polydispersity index.

Sample		PdI ± SD	Hydrodynamic diameter, nm ± SD	Area, % ± SD
**5 nm PtNPs**	alone	0.5 ± 0.06	22.96 ± 1.31<br>615.9 ± 73.4<br>3897.5 ± 284.96	67.03 ± 3.78<br>27.7 ± 1.91<br>5.23 ± 4.82
	+ 5 μM IDA	0.74 ± 0.01	171.7 ± 19.9<br>19.62 ± 1.7<br>4074 ± 539.75	55.93 ± 2.13<br>36.7 ± 1.45<br>7.33 ± 2.76
	+ 50 μM IDA	0.24 ± 0.03	539.73 ± 16.05	100
**30 nm PtNPs**	alone	0.07 ± 0.01	42.93 ± 0.36	100
	+ 5 μM IDA	0.07 ± 0.02	42.58 ± 0.43	100
	+ 50 μM IDA	0.15 ± 0.01	138.1 ± 34.45	100
**50 nm PtNPs**	alone	0.05 ±0.01	57.31 ± 1.35	100
	+ 5 μM IDA	0.06 ± 0.01	57.8 ± 0.44	100
	+ 50 μM IDA	0.13 ± 0.01	96.76 ± 0.56	100
**70 nm PtNPs**	alone	0.06 ± 0.03	87.4 ± 3.04	100
	+ 5 μM IDA	0.02 ± 0.01	90.24 ± 3.05	100
	+ 50 μM IDA	0.11 ± 0.02	133.97 ± 4.6	100

Table S3. **Data for the AFM analysis of platinum nanoparticles (PtNPs) alone and in combination with idarubicin (IDA).** SD – standard deviation.

	5 nm PtNPs		30 nm PtNPs		50 nm PtNPS		70 nm PtNPs	
	Alone	With IDA	Alone	With IDA	Alone	With IDA	Alone	With IDA
**Mean size (nm)**	5.4	1217.6	41.75	1927.8	66.63	1435.2	80.75	1600.25
**SD**	0.9	165.9	10.8	481.2	28.3	680.6	15.6	187.8

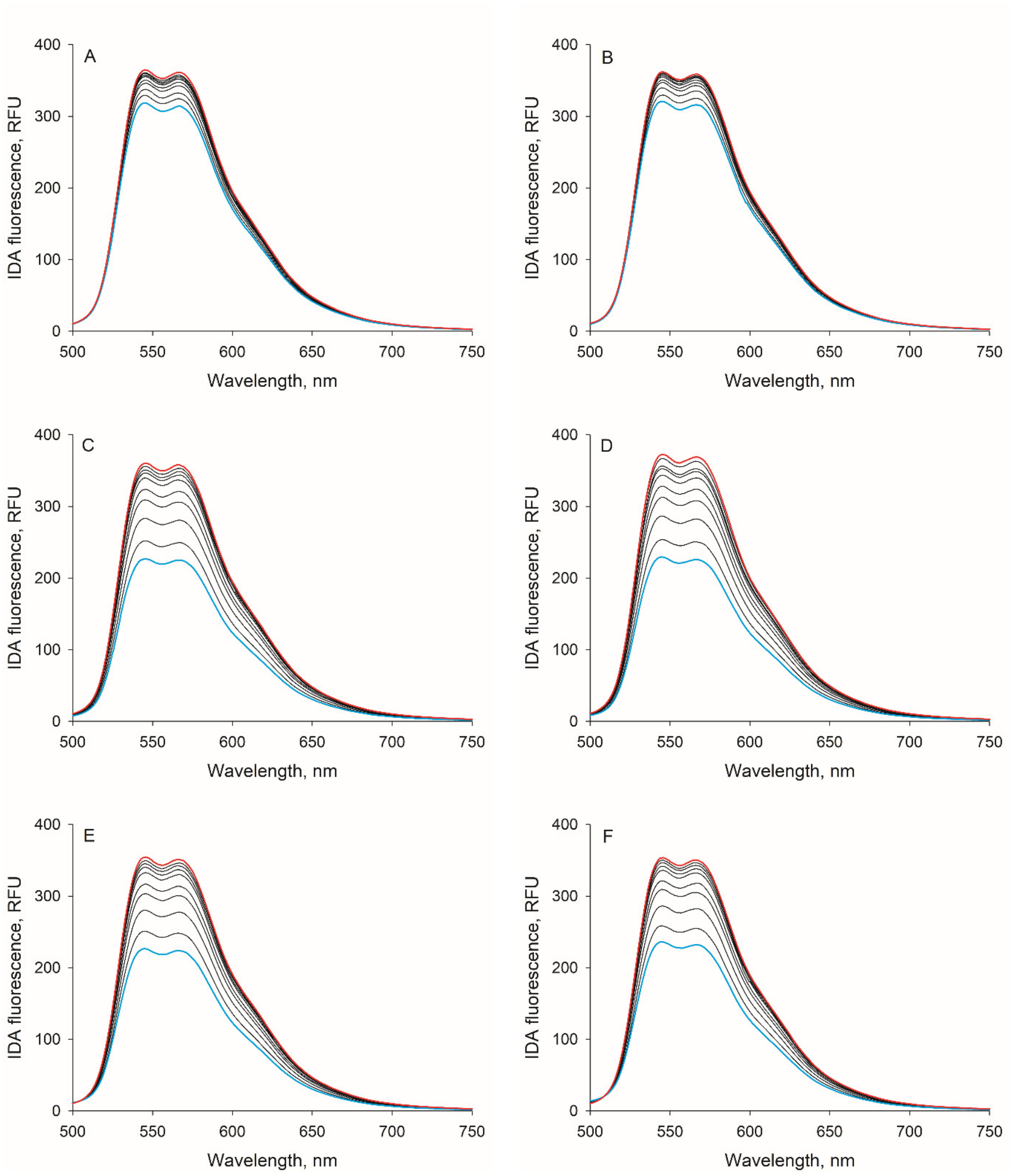

Figure S1. Changes in the fluorescence of idarubicin (IDA) upon titration with various sizes of platinum nanoparticles (PtNPs) or appropriate sodium citrate solution (buffer). (**A, B**) 2 mM or 4 mM sodium citrate, respectively, (**C – F**) 5, 30, 50, and 70 nm PtNPs, respectively. Red line – IDA alone, blue – IDA with the highest titrated volume of PtNPs or buffer. IDA concentration range: 37.2 – 29.3 µM, PtNPs concentration range 0 – 10.62 µg/mL.

Table S4. Fourier transform infrared spectroscopic (FTIR) characterization of idarubicin (IDA) alone and in combination with various sizes of platinum nanoparticles (PtNPs). The regions of significant changes in IDA's band were marked in red.

Chemical Bond	IDA 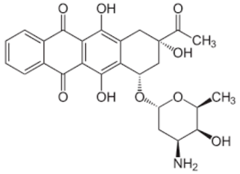	IDA<br>Lyophilised from sodium citrate solution	5 nm PtNPs with IDA<br>Complex 1	30 nm PtNPs with IDA<br>Complex 2	50 nm PtNPs with IDA<br>Complex 3	70 nm PtNPs with IDA<br>Complex 4
**O-H intermolecular bonded**	3578	–	–	–	–	–
**N-H stretching primary amine**	3535	3600 – 3400	3535	3535	3535	3535
**O-H intermolecular bonded**	3436, 3410, 3352	3417, 3350,	3436, 3410, 3357	3436, 3410, 3357	3436, 3410, 3357	3436, 3410, 3352
**C-H (CH_2) stretching alkane**	3072	3066	3066	3066	3066	3066
**O-H stretching alcohol intramolecular bonded**	2992, 2946, 2924, 2904	2979, 2924	2979, 2924	2979, 2924	2979, 2924	2979, 2924
**hydrogen-bonded dimers of ketone group**	2707, 2663, 2611, 2546	2542	2546	2546	2546	2546
**Asymmetric acid −C=O ketone**	1713	1713	1713	1713	1713	1713
**(benzoilo)-C=O amide**	1623	1621	1621	1621	1621	1621
**−C=C-**	1587, 1521	1587	1587, 1562	1587, 1562	1587, 1562	1587, 1562
**C-H (CH_3)**	1454, 1431, 1352, 1338, 1324	1454, 1349, 1321	1403 – 1380 1394	1403 – 1380 1398	1403 – 1380 1398	1403 – 1380 1401
**O-H bending alcohol**	1408, 1371	1402, 1375	1402, 1371	1402, 1371	1402, 1371	1402, 1371
**−C-O-C-**	1263	1264	1298	1281	1281	1281
**C-O stretching ester or −C-N- amine**	1225, 1205,	1231, 1204	1231, 1204	1231, 1204	1231, 1204	1231, 1204
	1165, 1147, 1133	1165, 1133	1165, 1133	1165, 1133	1165, 1133	1165, 1133
**O-H alcohol**	1117	1117	1117	1117	1117	1117
**C-O stretching**	1086	1082	1082	1082	1080	1078, 1063
**−C-O-C-**	1028, 1009, 977	1030, 1010, 986	1030, 1010, 986	1030, 1010, 986	1030, 1010, 986	1030, 1010, 986
**C=C bending alkene substituted**	950, 941	941	941	941	941	941
**strong C-H bending**	918, 886, 872, 853, 816,	916, 888, - 872, 815	916, 888, -, 872, 815	916, 888, -, 872, 815	916, 888, -, 872, 815	916, 888, -, 872, 815
**C=C bending**	798, 769, 729, 710	797, 767, 731	797, 767, 731	797, 767, 731	797, 767, 731	797, 767, 731

Table S5. Near infrared spectroscopic (NIR) characterization of idarubicin (IDA) and its complexes with various sizes of platinum nanoparticles (PtNPs). The regions of significant changes in IDA's band were marked in red.

**Chemical Bond**	**IDA**	**IDA**	**5 nm PtNPs with IDA**	**30 nm PtNPs with IDA**	**50 nm PtNPs with IDA**	**70 nm PtNPs with IDA**
	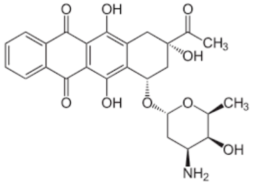	**Lyophilized from sodium citrate solution**	**Complex 1**	**Complex 2**	**Complex 3**	**Complex 4**
**O–H alcohol (RO–H), N–H amine**	6200 – 5500	6160 – 5340	6300 – 5300	6300 – 5300	6300 – 5300	–
**O–H assigned to molecular water, N–H combination, amine**	5300 – 4900<br>5149	5300 – 4900<br>5149	5300 – 4900<br>5092	5300 – 4900<br>5144	5300 – 4900<br>5146	5300 – 4900<br>5136
**C=O and C–O stretching, N–H/C–N, C–H aromatic C–H (aryl)**	4718, 4666	4718, 4666	4718, 4666	4718, 4666	4718, 4666	4718, 4666
**O–H/C–O, C–H starch (.C–H and CH_2), C–H (.C–H bending)**	4358	4358	4358	4358	4358	4358

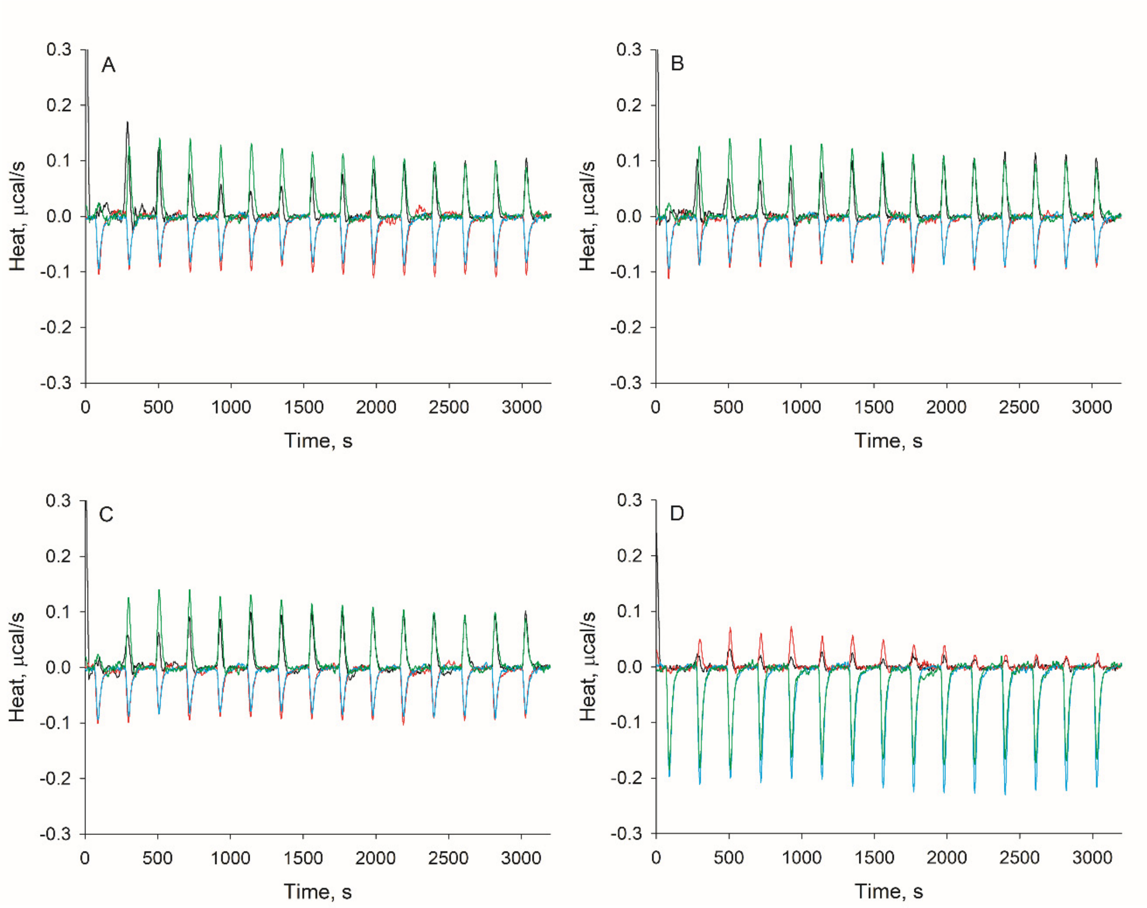

Figure S2. Thermograms showing microcalorimetric titrations of platinum nanoparticles (PtNPs) with idarubicin (IDA). (**A**) 5 nm PtNPs, (**B**) 30 nm PtNPs, (**C**) 50 nm PtNPs, (**D**) 70 nm PtNPs. Black – PtNPs with IDA, red – water with PtNPs, cyan – water with appropriate sodium citrate solution, green – IDA with appropriate sodium citrate solution.

Table S6. DSC data for idarubicin (IDA) samples combined with platinum nanoparticles (PtNPs) of different sizes. Numbers marked in red are of significant change with respect to lyophilised IDA.

	IDA lyophilised from sodium citrate solution	IDA with 5 nm PtNPs	IDA with 30 nm PtNPs	IDA with 50 nm PtNPs	IDA with 70 nm PtNPs
**Water loss T [°C]**	105.65	99.15	93.51	102.80	113.58
**ΔH [J/g]**	-17.88	-4.86	-3.01	-3.35	-33.37
**T_m [°C]**	185.66	185.95	185.00	185.43	186.29
**ΔH [J/g]**	-15.47	-19.48	-18.39	-20.59	-12.91
**T_d [°C]**	208.40	204.70	207.05	206.87	208.70
**ΔH [J/g]**	20.26	17.77	21.67	24.89	12.36

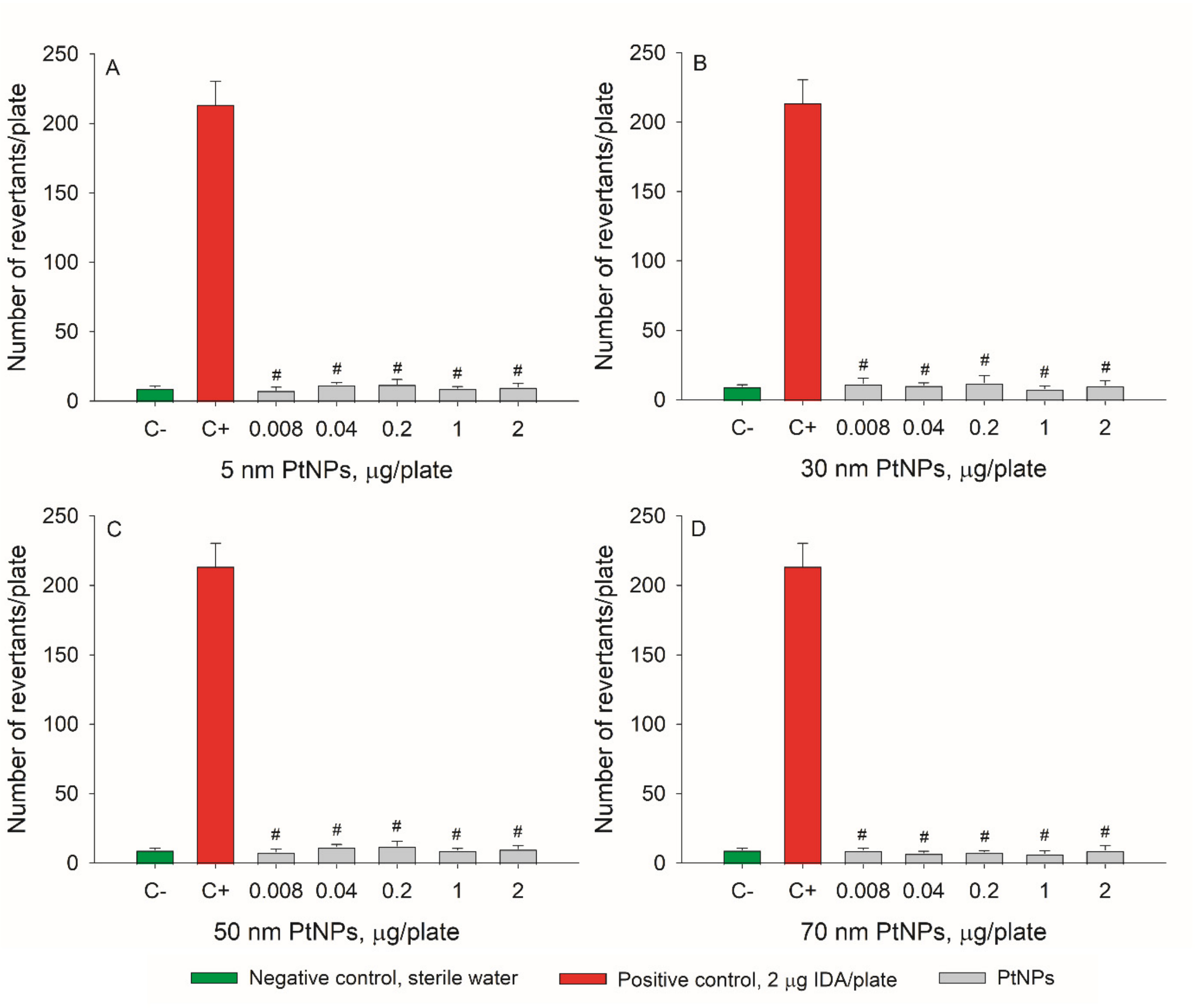

Figure S3. Mutagenicity of platinum nanoparticles (PtNPs) in *Salmonella enterica* serovar Typhimurium TA98 strain. (**A – D**) 5, 30, 50, and 70 nm PtNPs, respectively. C- negative control (sterile water), C+ positive control (2 µg IDA/plate). Results are reported as the average number of revertants per plate ± SD. #- significant difference from the positive control ($p < \alpha$; $\alpha = 0.05$).

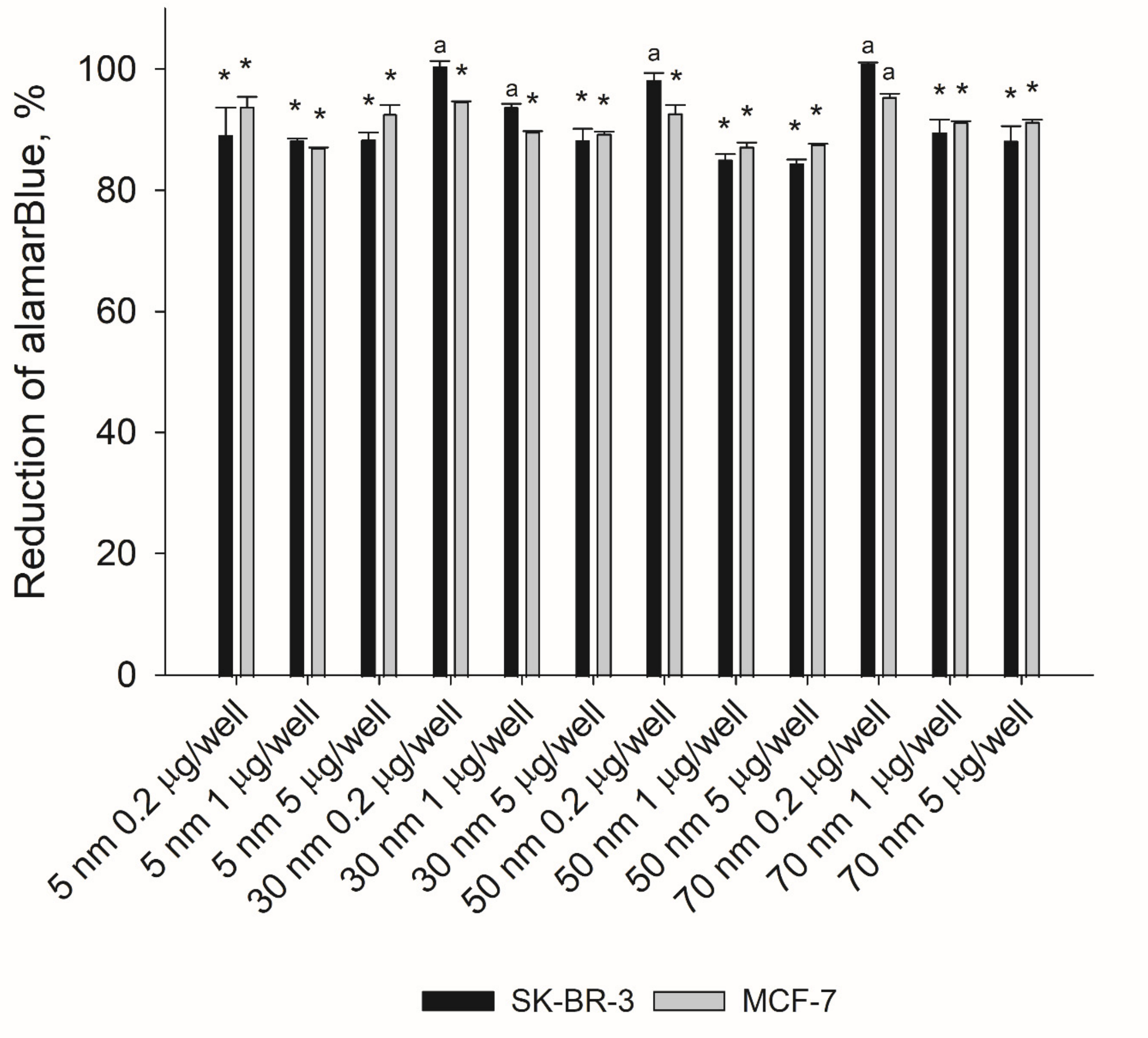

Figure S4. Anticancer activity of platinum nanoparticles (PtNPs) in the SK-BR-3 and the MCF-7 cell lines. Results are reported as the mean reduction of alamarBlue in regard to the untreated cells (negative control, 100% viability) ± standard deviation (SD). a – no significant difference, * - significant difference ($p < \alpha$, $\alpha = 0.05$).

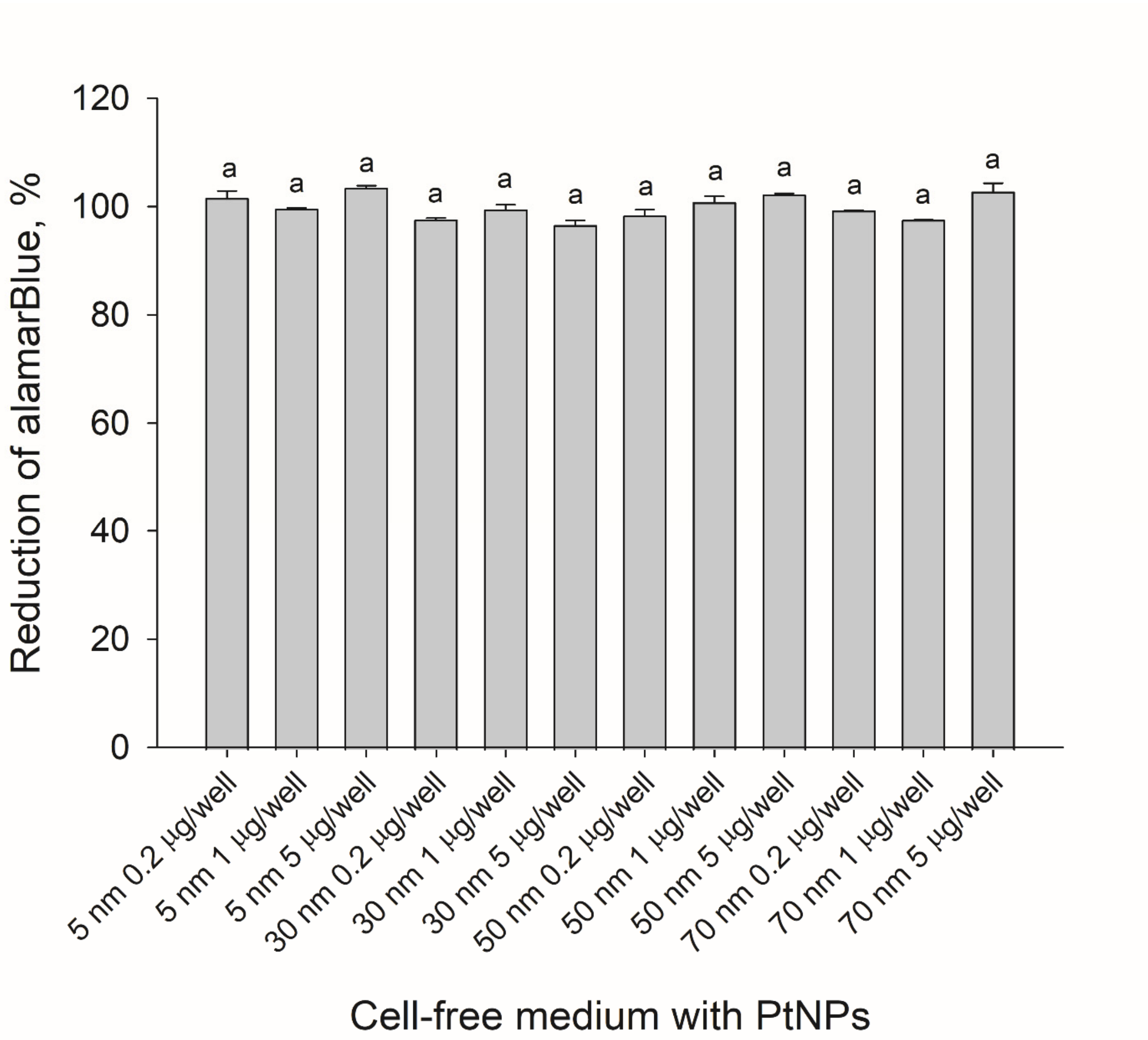

Figure S5. Cell-free interference assay. Incubation of cell-free medium with PtNPs and the addition of alamarBlue for 4h. Results are reported as the mean reduction of alamarBlue in regard to well with cell-free medium and alamarBlue only ± standard deviation (SD). a – no significant difference, * - significant difference ($p < \alpha$, $\alpha = 0.05$).
